# Co-acclimation of bacterial communities under stresses of hydrocarbons with different structures

**DOI:** 10.1038/srep34588

**Published:** 2016-10-04

**Authors:** Hui Wang, Bin Wang, Wenwen Dong, Xiaoke Hu

**Affiliations:** 1 Key Laboratory of Coastal Biology and Bioresource Utilization, Yantai Institute of Costal Zone Research, Chinese Academy of Sciences, Yantai, 264003, China

## Abstract

Crude oil is a complex mixture of hydrocarbons with different structures; its components vary in bioavailability and toxicity. It is important to understand how bacterial communities response to different hydrocarbons and their co-acclimation in the process of degradation. In this study, microcosms with the addition of structurally different hydrocarbons were setup to investigate the successions of bacterial communities and the interactions between different bacterial taxa. Hydrocarbons were effectively degraded in all microcosms after 40 days. High-throughput sequencing offered a great quantity of data for analyzing successions of bacterial communities. The results indicated that the bacterial communities responded dramatically different to various hydrocarbons. KEGG database and PICRUSt were applied to predict functions of individual bacterial taxa and networks were constructed to analyze co-acclimations between functional bacterial groups. Almost all functional genes catalyzing degradation of different hydrocarbons were predicted in bacterial communities. Most of bacterial taxa were believed to conduct biodegradation processes via interactions with each other. This study addressed a few investigated area of bacterial community responses to structurally different organic pollutants and their co-acclimation and interactions in the process of biodegradation. The study could provide useful information to guide the bioremediation of crude oil pollution.

Over one million tons of petroleum enters marine environments, originating from both natural (natural seeps of oil and natural gas) and anthropogenic sources every year^1^. Marine pollution by crude oil remains, a major threat to the marine environment and receives public and scientific attention^2^. Notably, disastrous oil spills, e.g., Lakeview Gusher oil spill (1910, United States), Gulf war oil spill (1990, Kuwait, Iraq), Deepwater Horizon oil spill (2010, Gulf of Mexico) and Penglai 19-3 oil spill (2011, Bohai Sea) poured tremendous amounts of crude oil into marine environment. Acute pollution incidents can lead to an immediate effect causing mass mortality and ecosystem services losses, as well as longer-lasting effects which can influence food webs^3^,^4^,^5^. Biodegradation is regarded as an important process for remediating oil polluted environments.

Crude oil is regarded as one of the most complex mixture of organic compounds, which is mainly composed of the saturated hydrocarbons, the aromatic hydrocarbons, and non-hydrocarbons (the resins and the asphaltenes)^6^. Because the saturated hydrocarbons constitute the largest fraction of the 20,000 chemical components of crude oil and aromatic hydrocarbons are very toxic and persistent, the biodegradation of hydrocarbons are acknowledged to be the most important process in bioremediation of crude oil contaminated environments^6^,^7^. Hydrocarbon-degrading bacteria are key players in this process. Since the first hydrocarbon degrading bacterium was isolated, at least 175 genera have been found to be capable of degrading hydrocarbons^6^,^8^. Bacterial strains from different phyla, including Proteobacteria, CFBs (Flexibacter-Cytophaga-Bacteroides), Actinobacteria, and Cyanobacteria have been successfully isolated from various environments and verified their efficient ability for degrading different hydrocarbons^9^,^10^,^11^,^12^,^13^. Especially, bacteria in genera *Alcanivorax*, *Cycloclasticus*, *Oleiphilus*, *Oleispira* and many other phylogenetic groups are well-acknowledged as ‘professional hydrocarbonoclastic bacteria’, since they always use hydrocarbons as priority carbon source^14^. Even some bacterial genera have wide spectrum for degrading different components of crude oil^15^,^16^, most of hydrocarbon-degrading bacteria are only capable of degrading a small range of hydrocarbons with similar structure, e.g. alkanes with different carbon chain length, or aromatic hydrocarbons with similar characteristics. A unique bacterial strain or genera could not degrade all components of the crude oil, due to the complexity of crude oil’s composition. Different bacteria dominate specific process of biodegrading hydrocarbons with different structures. For instance, bacterial genus *Alcanivorax* was regarded as the typical functional group specifically degrading strait-chain or branched alkanes^9^,^17^, while bacteria in the genus *Cycloclasticus* showed priority in degrading PAHs^18^,^19^.

Although preferences of different substrates by different hydrocarbon-degrading bacteria have been well-studied in laboratory by using pure cultures, little is known about functions of individual bacterial taxa and co-acclimations between different bacteria with degrading ability in the degrading process. McKew *et al*.^20^ tried to investigate the key bacterial taxa involved in aerobic degradation of specific hydrocarbons in a previous work. The results indicated that bacteria in the genera *Thalassolituns* and *Roseobacter* dominated the process of *n*-alkane degradation; bacteria in the genus *Alcanivorax* were responsible for the degradation of branched alkane, and *Cycloclasticus* was the dominant genus in PAHs degradation. This study provided preliminary data about partition of different petroleum hydrocarbons by microbial population. Further investigations could be conducted on revealing individual functions of different bacterial taxa and interactions between different microbes in the process of biodegradation. Here, a series of microcosms were setup with addition of petroleum hydrocarbons with different structures, to investigate 1, successions of bacterial communities under stresses of different hydrocarbons; 2, functions and contributions of different bacterial taxa in each steps of hydrocarbon-degrading; 3, co-acclimations between different bacterial phylotypes or between different functional groups. This study could provide a better understanding of co-acclimation of functional bacteria in the process of biodegrading hydrocarbons.

## Results

### Biodegradation of different hydrocarbons

After the four times domestication with 10 d time’s interval, bacterial communities in sediments showed great degrading efficiency to different hydrocarbons (Supplementary Fig. S2). Biodegradation efficiencies detected at the last domesticating period (30–40 d) for tetradecane, pristane and pyrene were 66.86%, 68.40%, and 74.69%, while higher values were obtained for hexacosane and phenanthrene, which could reach up to 95.67% and 99.62% separately.

### Sequencing data and taxonomic identification

Miseq High-throughput sequencing of 16S rRNA gene amplicons yielded 1,319,248 raw reads. After quality filtering and normalizing, 1,237,359 sequences were selected for further analysis. The numbers of sequences used for duplicate samples of original sediment were 3348 and 3387, while numbers for all other samples ranged from 4964 to 6423. This generated totally 4750 OTUs. Each duplicate sample had a mean of 1532 OTUs (AVEDEV = 101). Rarefaction curve analysis (Supplemental Fig. S3) indicated that sequencing captured the majority of the bacterial diversity in the samples. These OTUs could be identified to different taxonomic level, which yielded 206 genera, 179 families, 123 orders, 71 classes and 31 phyla. A small amount of OTUs were identified as Archea (0.03% of all sequences). At the phylum level, members of Proteobacteria, Bacteroidetes, and Actinobacteria dominated the bacterial group with proportions of 67.84%, 15.95%, and 7.41% respectively. All other phyla contributed less than 5% of total sequences. The most common classes were Gammaproteobacteria (36.06%), Alphaproteobacteria (25.51%), Flavobacteriia (9.87%), and Actinobacteria (6.36%). Dominant families were Pseudomonadeceae (19.79%) and Erythrobacteraceae (12.49%), and the dominant genus was Pseudomonas (14.79%). 1.59% of all sequences were assigned to known species.

### Successions of bacterial communities

Significant temporal variations of bacterial communities were found during the domestication period, as well as dramatically different responses of bacterial communities to structurally different hydrocarbons were also observed in this study. By comparing bacterial phylotypes which showed dramatic changes at the third domestication phase and those at the fourth domestication phase, we found that bacterial communities reached a relatively static status after 30 d incubation (Supplemental Fig. S4). For example, in the treatment of tetradecan, 22 bacterial groups in the genus level showed significant changes after 30 d and 40 d domestication. 77.27% (17 of 22) bacteria groups showed same or similar variation trend (including increase, decrease, or disappear), while only 22.73% of all bacterial groups were only found, in one of the two domestication periods. A similar phenomenon was also observed for bacterial communities under stresses of other four hydrocarbons. 68.75%, 77.28%, 75.00% and 90.48% of bacterial communities (F-test < 0.1) in treatments of pristane, hexacosane, pyrene and phenathrene showed same or similar variation trend after 30 d and 40 d domestication. This meant that structures of bacterial communities were stable after 30 d co-incubation with different hydrocarbons.

Although Alphaproteobacteria, Gammaproteobacteria, and Actinobacteria were dominant in all Miseq sequencing data, obvious variations of bacterial communities at phyla-level (including classes in proteobacteria) were detected under stresses of different hydrocarbons (Supplemental Fig. S5). For instance, Alphaproteobacteria became dominant groups after 10 d co-incubation with tetradecane, and accounted for up to 60% of total sequences after 40 d domestication. For hexacosane and pyrene, the dominant bacterial groups were Gammaproteobacteria instead of Alphaproteobacteria, which accounted upto 60–70% of total sequences. In the treatments with addition of pristane, the above bacterial groups contributed, almost equally to the total bacterial sequences (32.40% for Alphaproteobacteria and 31.30% for Gammaproteobacteria). When phenathrene was considered, Bacteriodetes (24.48% of total bacterial sequences) became the third most dominant group, following with the pre-mentioned two proteobacterial groups. Common feature for bacterial communities of all five different treatments could be found when Deltaproteobacteria and Epsilonproteobacteria were considered. These two proteobacterial classes almost disappeared after 40 d co-incubation with different hydrocarbons, although proportions of these two groups could research up to 13.22% and 14.98 of total sequence in original sediments, respectively.

**Figure 1:**
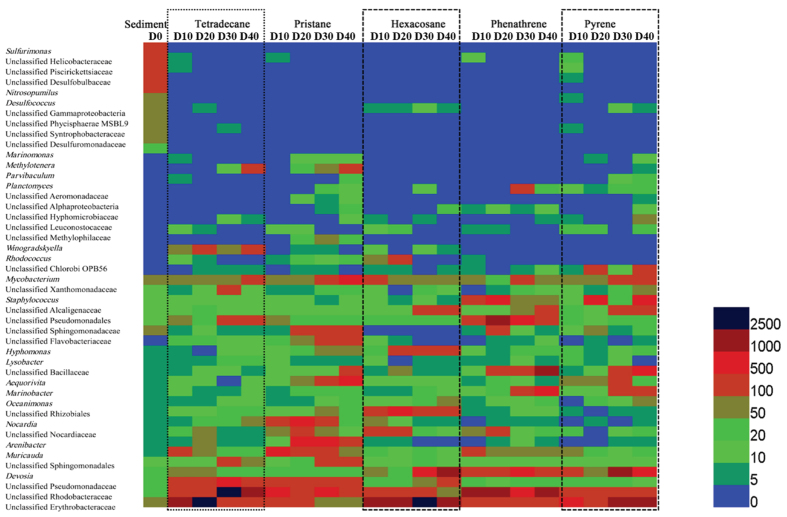
Heatmap profile showing the relative abundance of different bacterial phylotypes in genus level. Number for different color of the column bar represents sequencing numbers for different phylotypes. Sequencing numbers at 0 d, 10 d, 20 d, 30 d, and 40 d of each hydrocarbon treatment were listed from left to right labeled as D0, D10, D20, D30, D40. Sequencing numbers were generated from mean value of duplicated samples.

To analyze successions of bacterial communities in depth, a heatmap profile was generated based on abundance of sequences at genera level (Fig. 1). In accordance with results at phyla level, some groups affiliated into Deltaproteobacteria (*e. g.* unclassified groups in families of Desulfobulbaceae, and Desulfuromonadaceae), and Epsilonproteobacteria (*e. g. Sulfurimonas* and an unclassified group in Helicobacteraceae) were abundant in sediment samples, but could hardly be found after co-incubation with five hydrocarbons. Contrarily, some other groups (e. g. *Pseudomonas* in Gammaproteobacteria and an unclassified group in the family of Erythrobacteraceae in Alphaproteobacteria) showed significant increase after domestication in all five treatments. When different treatments were considered, obvious variations of dominant bacterial phylotypes were discovered. Three unclassified groups in families Erythrobacteraceae, Xanthomonadaceae, and Rhodobacteraceae, dominated bacterial communities in the treatment with addition of tetradecane. Sequences affiliated with the genus *Pseudomonas* increased in the first 20 d, but decreased after 20 d and kept steady at the two last domestication phases. When pristane was added, sequences in genera *Marinobacter*, *Hyphomonas*, *Muricauda*, Devosia, and the two unclassified groups in families Erythrobacteraceae and Xanthomonadaceae, became dominant groups. For the treatment of hexacosane, two Gammaproteobacterial groups, *Pseudomonas* and an unclassified group in the family of Pseudomonadaceae showed great superiority compared with all other phylotypes. The two dominant groups changed to *Aequorivita* and the unclassified groups in the family Erythrobacteraceae when phenanthrene was added as a stress. Bacterial sequences in the two genera *Pseudomonas* and *Aequorivita* and other two unclassified groups in families Alcaligenaceae and Pseudomonadaceae dominated bacterial communities after domestication with addition of pyrene.

**Figure 2:**
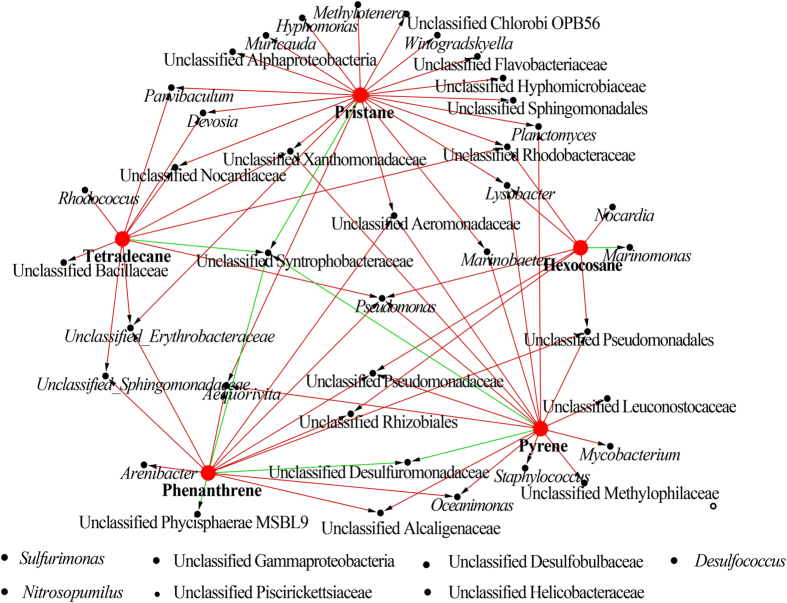
Network profile indicating responses of different bacterial phylotypes to hydrocarbons with different structures after 40 d co-incubation. Red dots represented different hydrocarbons while black dots represented bacterial phylotypes showing significant differences (F-test < 0.09) between that in sediment and in 40 d samples. Red solid lines indicated that sequencing number increased in 40 d samples. Green solid lines indicated that sequencing number decreased in 40 d samples.

The network constructed by using bacterial phylotypes with significant changes (F-test < 0.1) and different hydrocarbons indicated varied responses of bacteria to different stresses (Fig. 2). Some bacterial phylotypes only showed dramatic responses to one single hydrocarbon. For instance, bacterial sequences in the genus *Nocardia* increased dramatically (81.6 times) after 40 d co-incubation with hexacosane, while sequencing numbers of the other genus *Marinomonas* decreased 93 times compared to the numbers in sediment samples. Meanwhile, some bacterial groups responded to two or more different hydrocarbons. For example, bacteria in genera *Parvibaculum* and *Devosia* showed greatly increasing rate with stresses of two *n*-alkanes, tetradecane and pristane, while bacteria affiliated into the genus *Oceanimonas* increased dramatically when the two PAHs were applied. Bacteria affiliated into the genus *Pseudomonas* showed significant increase in treatments with addition of tetradecan, hexacosane, phenethrene, and pyrene. The unclassified bacterial group in the family Syntrophobacteraceae responded negatively to pristane, tetradecane, phenathrene and pyrene, except for the C_26_ alkane. Bacterial phylotypes in genera *Sulfurimonas*, *Nitrosopumilus*, and *Desulfococcus*, and some unclassified groups in families Desulfobulbaceae, Helicobacteraceae, and Piscirickettsiaceae, and an unclassified group in the class of Gammaproteobacteria, which were aligned at the bottom of the network (Fig. 2), showed the dramatically negative responses to all five hydrocarbons. These bacterial groups could hardly be found in all five treatments after 40 d co-incubation.

### Functional bacteria involved in metabolic pathways

**Figure 3:**
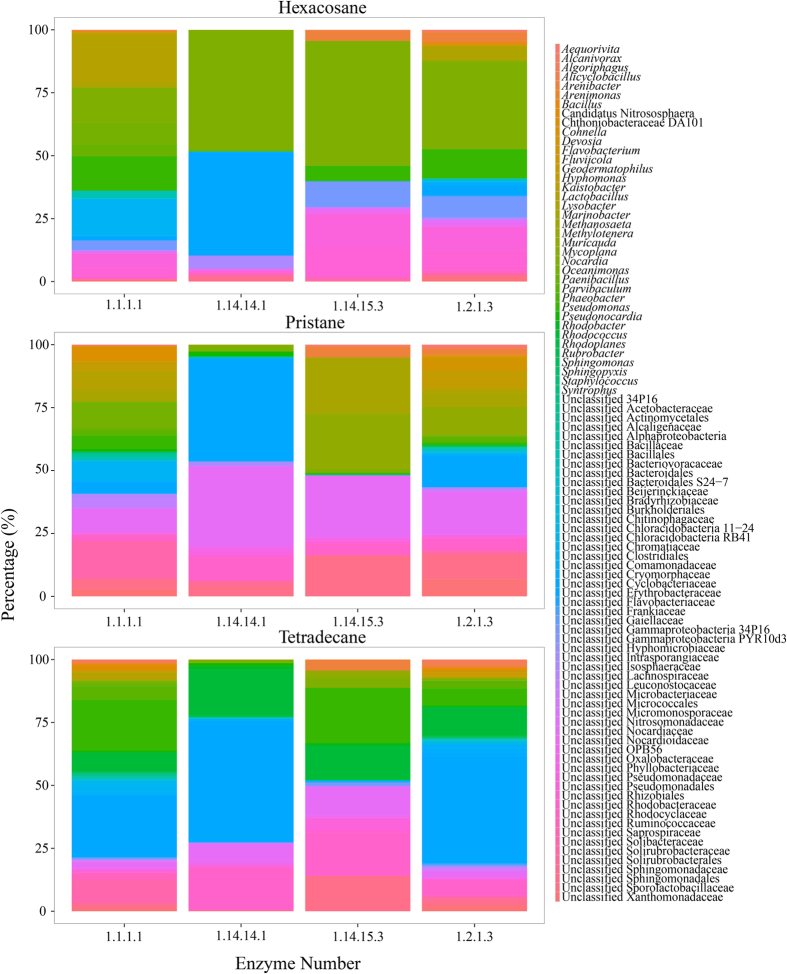
Bacterial phylotypes participated in different steps of alkane degradation pathway. Enzyme numbers on X axis represented enzymes involved in the different steps of alkane degradation.

Functions of different OTUs and proportions of functional bacterial groups participated in each step of biodegradation were predicted by using KEGG and PICRUSt. There were four steps for biodegrading alkane to *α*/*ω*-hydrocy fatty acid which could be easier degraded via the fatty acid degradation pathway (Supplemental Fig. S6). Four different enzymes were involved into the four steps of the primary and important pathway, including 1-monooxygenase (EC: 1.14.15.3), alcohol dehydrogenase (EC: 1.1.1.1), aldehyde dehydrogenase (EC: 1.2.1.3) and unspecific monooxygenase (EC: 1.14.14.1). 1-monooxygenase could participate in the first and the fourth steps, and the other three only participate in one step during the metabolic process. Functional genes encoding for four enzymes were obtained by PICRUSt by using greengene accession number of dominant bacterial OTUs. Even three alkanes shared the same pathway, bacterial communities involved in degrading processes of different alkanes varied dramatically (Fig. 3). The unclassified bacterial group in Erythrobacteraceae was proved to be dominant group in three out of total four steps of tetradecane biodegradation, including the second, third and fourth steps. *Pseudomonas*, *Rhodococcus* and two unclassified phylotypes in families Rhodobacteraceae and Sphingomonadales were four main groups carrying genes encoding 1-monooxygenase, when tetradecane was degraded. But for pristane, the main bacterial phylotypes carrying 1-monooxygenase related genes changed to *Marinobacter*, *Muricauda*, and two unclassified phylotypes in families Nocardiaceae and Sphingomonadales. For the other three steps in pristane degradation, unclassified phylotypes in families Nocardiaceae, Erythrobacteraceae and Solibacteraceae were regarded as principal groups carrying functional genes encoded with enzymes EC: 1.2.1.3, EC: 1.14.14.1 and EC: 1.1.1.1, respectively. When hexacosane was applied as a stress, bacteria in the genus *Nocardia* dominated all four steps of biodegradation. The unclassified phylotype in family Erythrobacteraceae also accounted for a considerable proportion (41.07%) for the gene encoded with the unspecific monooxygenase in the fourth step, which was 6% less than the proportion of *Nocardia*.

**Figure 4:**
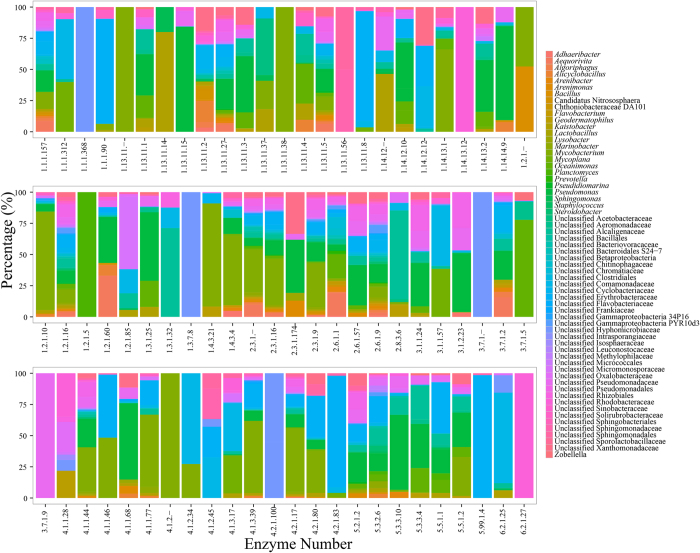
Bacterial phylotypes participated in different steps of pyrene degradation pathway. Enzyme numbers on X axis represented enzymes involved in the different steps of pyrene degradation.

**Figure 5:**
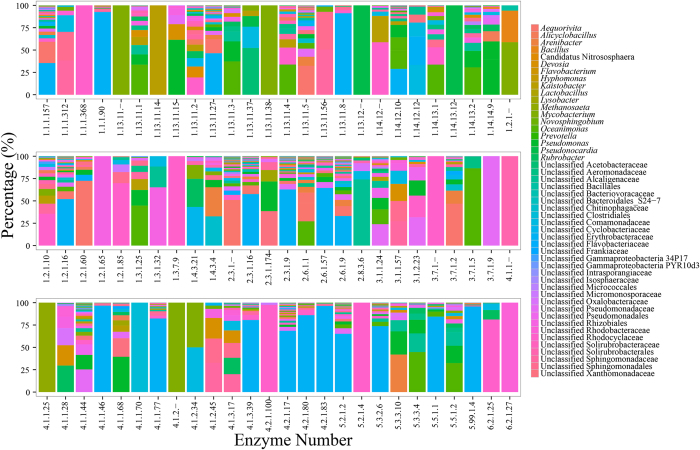
Bacterial phylotypes participated in different steps of phenathrene degradation pathway. Enzyme numbers on X axis represented enzymes involved in the different steps of phenathrene degradation.

The “Xenobiotics biodegradation and metabolism” list on KEGG website (http://www.kegg.jp/kegg/) suggested pathways for pyrene and phenanthrene degradation. Pyrene and phenanthrene could be biodegraded to 3,4-dihydroxy-phenanthrene (pathway name as rn00624) which could be further metabolized into tricarboxylic acid cycle (TCA cycles) via “Naphthalene degradation (rn00626)”, and “Benzoate degradation (rn00362)” or “Tyrosine metabolism (rn00350)”. Most of functional genes encoded with enzymes in these four pathways were successfully predicted in bacterial communities treated by pyrene and phenathrene (Supplemental Fig. S7). Since four enzymes with EC number of 1.14.-.-, 1.13.11.-, 4.1.1.- and 1.3.-.- of the total six enzymes involved in the process of degrading pyrene to 3,4-dihydroxy-phenanthrene lacks further study and did not have functional gene numbers, bacterial communities potentially participated in the initial several steps of pyrene degradation were failed to be predicted. Genes encoded with 84 enzymes were predicted, which involved in 92 catalyzing reaction of total 107 reactions in the process of transferring pyrene to TCA cycle. As the results suggested, most reactions were performed by several bacterial phylotypes, while some reactions were conducted by only one bacterial group (Fig. 4). For example, *Mycobacterium* was regarded as the unique functional bacterial group in four key reactions catalyzed by enzymes with EC numbers of 1.13.11.-, 1.13.11.38, 4.1.1.25, and 4.1.2.-. An unclassified group in the family of Hyphomicrobiaceae in Alphaproteobacteria was exclusive in six different reactions mainly involved in the two pathways of “Benzoate degradation” or “Tyrosine metabolism”. Varied bacterial groups were dominant in different reactions employing more than one bacterial phylotypes. Functional genes predicted from bacteria in the genus *Pseudomonas* could encode with 43 different enzymes in the process of pyrene degradation and showed priority in reactions catalyzed by 35 of these enzymes (>10% of total predicted functional genes for each reaction). Bacteria in the genus *Mycobacterium* were found to contribute to reactions catalyzed by 39 different enzymes, including the four enzymes mentioned above. This group of bacteria were abundant (>10%) in almost of these reactions, except for the reaction catalyzed by 4-hydroxyphenylpyruvate dioxygenase (EC: 1.13.11.27), which was mainly contributed by bacteria in the genus *Pseudomonas*. Bacteria in the genus *Oceanimonas*, and three unclassified groups in families of Alcaligenaceae, Comamonadaceae, and Erythrobacteraceae were also found to be abundant in different catalyzing reactions. Contributions of different bacterial phylotypes to reactions in the process of biodegrading the other PAH, phenanthrene were exhibited in Figure 5. Similar to the biodegradation of pyrene, some reactions were predicted with only one functional bacterial group (Fig. 5). These bacterial groups included *Mycobacterium*, *Kaistobacter*, *Methanosaeta*, *Pseudonocardia* and unclassified phylotypes in families Rhizobiales, Rhodobacteraceae, Rhodocyclaceae, Pseudomonadaceae, Clostridiales and Solirubrobacterales. Besides these exclusive groups, some bacterial phylotypes, *e.g*. *Pseudomonas*, *Oceanimonas*, and the unclassified group in the family of Erythrobacteraceae, showed great priority in catalyzing different reactions.

### Functional groups in networks

Bacterial communities with significant correlations during the domestication period were applied to construct networks which could demonstrate the co-acclimation of functional bacteria. Five complex networks were generated for five different treatments using different hydrocarbons respectively (Supplemental Fig. S8–S12). All five networks showed tight interactions between different bacterial phylotypes, bacterial phylotypes with similar variation tendency which could be grouped together and create numerous “functional groups”. These functional groups could be separated from the whole network as “subnetworks” by executing “cytocluster” in the software of cytoscape. Combing predicated functions for each bacterial phylotype, these functional groups were annotated with different functions (Supplemental Tables S1–S5). For example, two functional groups from hexacosane-network were listed in the Supplemental Table S3. The first functional group included 14 different bacterial phylotypes. Five phylotypes including *Nocardia*, *Aequorivita*, Unclassified Frankiaceae, Unclassified Micrococcales, Unclassified Intrasporangiaceae, carrying degradation related genes were detected in this group. As mentioned above, bacteria in the genus *Nocardia* contributed greatly in the process of hexacosane degradation. This indicated that *Nocardia* could be the key element of the “functional group” and make the group become a “degrader” in the process of hexacosane degradation. Five interested groups were selected from the network generated for the treatment with addition of pyrene. These functional groups included different numbers of bacteria phylotypes (3–14). The “functional group” 3 involved in all the four steps of biodegradation of pyrene, which mainly attributed to the key member of the group, *Mycobacterium*. The other four groups included different bacterial phylotypes contributed to different steps of biodegradation. For example, the “functional group” 4, which included several different phylotypes carrying degradation related genes, contributed mainly in the three steps of naphthalene, tyrosine and benzoate degradation/metabolism, but not the step of PAHs biodegradation.

## Discussion

Petroleum hydrocarbons were found to have two entirely different effects on bacterial communities. On one hand, each compound could be potentially being a carbon or energy source for some bacteria which could utilize these compounds or their metabolites for growth. On the other hand, the same compound could be toxic for other bacteria. Thus, compositions of bacterial communities could be, significantly influenced when petroleum hydrocarbons are introduced to environments. For example, the Deepwater Horizon Oil Spill happened on 20 April 2010, leaked over four million barrels of oil into the Gulf of Mexico^21^. Dramatic successions of bacterial communities were observed after the oil spill^22^,^23^,^24^,^25^,^26^. In this study, by addition of individual hydrocarbon in microcosms, we were able to reveal the different successions of bacterial communities under stresses of varied hydrocarbons. Some bacterial taxa were proved to be generalists being capable of degrading structurally different hydrocarbons including *n*-alkanes, branched alkane and PAHs. The known lists of these generalists included *Roseobacter*, *Acinetobacter*, *Marinobacter*, *Pseudomonas* and *Rhodococcus*^27^,^28^,^29^,^30^,^31^. The results of this study indicated that an unclassified group in the family of Erythrobacteraceae in Alphaproteobacteria could be added into this list. Sequences belonging to this phylotype showed obvious increase under stresses of all five different hydrocarbons. Although there is still no literature supporting that this group could directly degrade hydrocarbons, it is believed this group of bacteria is involved in the pathways of biodegradation of hydrocarbons, considering the bacteria behavior in this study. In contrast, bacteria affiliated into the genus *Sulfurimonas* and three unclassified groups in families of Desulfobulbaceae, Desulfuromonadeceae and Helicobacteraceae could not be detected after 40 d co-incubation with five different hydrocarbons. The first three groups were well-acknowledged to participate in the geochemical cycle of sulfur^32^. This indicated that the sulfur recycling might not be essential for hydrocarbon degradation, or hydrocarbons and their metabolites were toxic to these bacterial groups. Bacteria genera *Nocardia* and *Devosia* were observed to be mainly positively responded to *n*-alkanes and branched alkanes. The genus *Nocardia* was previously implicated in long-chain *n*-alkanes and functional genes (*alk*B, *rub*A3, *rub*A4, and *rub*B gene) were successfully detected^33^,^34^. After being co-incubated with hexacosane, sequences encoding with *Nocardia* increased more than 80 times. Also, bacteria in this genus could degrade pristane which was regarded to be more recalcitrant to degradation and was always used as a slowly biodegradable biomarker^35^. The results indicated that the branched alkane was efficiently degraded (68.40%) in our microcosms. The culture-based experiment also supported that most of cultured bacterial colonies under the stress of pristane were affiliated into the genus *Nocardia* (Supplemental Table S6). Bacteria in the genera *Hyphomonas* also showed dramatic increase only with the treatment with pristane. For biodegradation of PAHs, the bacterial taxa of the genus *Aequorivita* was firstly reported to be related to the degrading process. The genus was proposed as a novel genus on 2002^36^, but functions of bacteria in this genus lacked of investigation. Data from here could provide a hint of the potential functions of the genus. Besides the bacteria taxa, some traditionally recognized PAHs degraders were also detected, including bacteria in genera *Pseudomonas*, *Mycobacterium*, and *Oceanimonas* and in families Alcaligenaceae, Pseudomonadaceae, Pseudomonadales and Erythrobacteraceae.

Analysis of the bacterial communities’ compositions, could reveal successions of bacterial communities and provide basic ideas about responses of bacterial communities to environmental changes, but could not provide direct evidence of potential functions for different bacteria. At first glance the bacterial communities used in this study, were able to find that bacterial taxa behave dramatically differently under stresses of different hydrocarbons, and different bacterial taxa dominate in differing degrading process. But detailed metabolic and functional profiles should be investigated by applying new approaches. High-throughput sequencing based on 16S rRNA gene could afford great magnanimity of sequences, which facilitate deep recognition of bacterial communities^37^. KEGG could provide pathways for biodegradation of different organic compounds and genetic and enzymatic information of functional bacteria involved in these processes^38^. PICRUSt was recently developed to bridge High-throughput sequencing for 16S rRNA gene and KEGG database^39^. The computational approach could predict functional composition of a metagenome by using 16S rRNA gene data and a database of reference genomes. Combining these three tools, Bier *et al*.^40^ investigated responses of bacterial communities to a gradient of alkaline mountain top, mine drainage and found that functional capacity predicted with PICRUSt correlated with mining in 3 of 43 KEGG Orthology groups. Here, by applying these tools, we could analyze the individual function of different bacterial taxa in the process of biodegradation. *n*-Alkanes and branched alkane were easily metabolized to *α*/*ω*-hydrocy fatty acid by four steps of catalytic reactions. Predicted bacterial taxa involved in the different steps of biodegradation, that uses different hydrocarbons were also varied which is consistent with successions of bacterial communities. For example, bacteria in the genus *Nocardia* were predicted to be involved in all four steps of hexacosane degradation, this is believed to play major role in the degrading process. Most of the functional genes involved in metabolic pathways of the two PAHs were also successfully predicted, while some key functional genes in the pathway of pyrene degradation were missing. For example, genes encoding pyrene dioxygenase catalyzing, the first step of pyrene degradation from pyrene to *cis*-4,5-Dihydroxy-4,5-dihydropyrene was not detected. Two possibilities were considered to account for the failure of catching these functional genes. The first being, cultured bacteria that is capable of degrading pyrene is still not well-exploited and the metabolic mechanism of pyrene degradation is far from well-known. Although some bacterial isolates were proven to grow on or mineralize pyrene^41^,^42^,^43^,^44^,^45^, the first complete pathway for catalyzing pyrene to TCA cycle was accomplished till 2007^46^. The lack of cultured functional bacteria and genetic annotation could cause numerous of functional bacteria in the predicted community mismatch the existing KEGG database. The second being, KEGG provided three potential pathways for pyrene degradation, but two of them were not explained in depth. Further, there might be more pathways that could exist in nature. This may cause the KEGG database not to contain all the potential genes functions involved in the degradation. Thus, the imperfect KEGG database may miss many functional genes. To solve the above two problems, more bacterial strain degrading pyrene should be isolated and genome should be well-annotated.

By analyzing functional genes, we could find that different bacteria might involve into different steps of degradation of PAHs. This indicated that interaction or co-acclimation might be necessary for most functional bacteria to perform the degradation. Interactions among bacterial phyloypes within a particular community, and their co-acclimation that reply to a specific environment change are always key research points in the area of microbial ecology. By constructing network for bacterial communities, these behaviors could be well elucidated^47^,^48^. Here, networks for bacterial communities under stresses of different hydrocarbons were constructed and analyzed. The results indicated that bacteria in a particular community interacted with each other consistently helping different bacterial species co-acclimated to the environmental stresses. Most of the functional bacteria only dominated in one or several steps of biodegradation. The cooperation between different bacterial species or functional group was necessary for mineralizing hydrocarbons. Bacteria affiliated into the phylotype of unclassified Erythrobacteraceae were dominant in the subnetwork 1 (Supplemental Table S5), and they could participate in three out of four major processes of pyrene degradation except the “PAH degradation” pathway which metabolizing pyrene to 3,4-dihydroxy-phenanthrene. This process could be completed by cooperation with bacteria affiliated into the group “Unclassified Pseudomonadaceae” in the subnetwork 2, bacteria in the genus ‘*Mycobacterium*’ in the subnetwork 3 and ‘*Kaistobacter*’ in the subnetwork 5 (Supplemental Table S5). Except for co-acclimation between functional groups, functional bacteria also could cooperate in a special functional group. Typically, five bacterial taxa in sub-network 5, played great roles in varied processes of pyrene degradation. Individual bacterial taxa might not be able to degrade pyrene sufficiently, while interactions between different bacterial taxa could stimulate the degradation. The results here could provide useful information for constructing bacterial consortium used for bioremediation of hydrocarbons and crude oil.

Some functional bacterial taxa were found to be capable of complete degradation of individual hydrocarbons. Coincidence with pre-mentioned successions of bacterial communities, bacteria in the genus *Nocardia* successfully involved in all four steps of metabolizing hexacosane to *α*/*ω*-hydrocy fatty acid. Meanwhile, the results indicated that bacteria in the genus ‘*Mycobacterium*’ contributed greatly for functional genes in all four processes of metabolizing pyrene to TCA cycle. This demonstrated that these two strains could perform the biodegradation process individually which were supported by metabolite analysis based on cultured strains^33^,^34^,^46^. But these two bacterial taxa which are grouped together with other bacteria could be with or without degrading ability in networks. This indicated that co-acclimation was still necessary for these functional bacteria. Co-acclimation and interactions in a special community occurred not only between “functional bacteria” with degrading ability who showed direct effect on hydrocarbons, but also between “functional bacteria” and bacteria without degrading ability^1^,^6^,^49^. A more integrated microbial network could be considered in future work which included both microorganisms that directly attack hydrocarbons (the primary oil degraders) and other components indirectly acted on hydrocarbons^6^. The network in broader sense should include more elements besides ones directly attack bacteria, for example, surfactant producing microorganism, mineral weathering microorganism, nutrient recycling microbes, phages/viruses, and protozoa. Although these microorganisms did not attack hydrocarbons directly, they could interact with “functional bacteria” to promote/inhibit the degrading process. Considering all these elements, the ecological network could deeply recognize species or processes that influenced hydrocarbon degradation *in situ*.

## Conclusion and Perspectives

In this study, we successfully revealed successions of bacterial communities and co-acclimation between different functional bacterial taxa under stresses of hydrocarbons with different structures. Bacteria in an unclassified group in the family of Erythrobacteraceae was proposed as a new generalist for degrading different hydrocarbons, while bacteria in the genera *Hyphomonas* and *Aequorivita* were found to be mainly responsible for stresses of pristane and PAHs respectively. Based on the network construction and functional prediction by KEGG database and PICRUSt, we could also demonstrate that functional bacteria co-acclimated to the changing environment, and conducted biodegradation via interactions with each other. More questions need to be addressed in the future research. For example, how would bacterial community responded to multi-hydrocarbons or crude oil? What are their individual roles in the process of degrading mixtures of hydrocarbons? Furthermore, broader networks including not only functional bacteria, which directly attack hydrocarbons should be considered as they recognize complex interactions between different microorganisms. And these networks should be verified via reconstruction of networks using cultured bacteria, which test key functional genes using RT-qPCR, *etc*. In all, this study provided preliminary but important data on interactions between bacteria in special bacterial communities and their co-acclimations, for changing environments. It could help accelerate related research into revealing more interactions between varied microorganisms.

## Materials and Methods

### Hydrocarbons and sediments

Five hydrocarbons with different structures were applied in this study (Supplemental Fig. S1), including two *n*-alkane (tetradecane, C_14_H_30_ and hexacosane, C_26_H_54_), a branched alkane (pristane, C_19_H_40_), and two PAHs (phenanthrene, C_14_H_10_ and pyrene, C_16_H_10_). Minimal salt medium (MSM) consisted of 1000 mg/L (NH_4_)_2_SO_4_, 800 mg/L Na_2_HPO_4_, 200 mg/L KH_2_PO_4_, 200 mg/L MgSO_4_•7H_2_O, 5 mg/L FeCl_3_•3H_2_O, 1 mg/L (NH_4_)_6_Mo_7_O_24_•4H_2_O, 100 mg/L CaCl_2_•2H_2_O, and 20 g/L NaCl. Different hydrocarbons at a final concentration of 400 mg/L were supplemented into MSM medium for further experiments. Sediments used in this study were collected from Bohai sea (Latitude 37°, 40′, 20″ N; Longitude 120°, 20′, 30″ E), north of China. Subsurface sediments were collected by using bottom sediment grab sampler and transferred to the laboratory immediately for further analysis.

#### Experimental setups

Aliquots (10 g) of sediments were inoculated into 100 ml MSM solutions and co-incubated with different hydrocarbons at 170 rpm, 25 °C. Ten milliliters of mixed liquid culture (including sediments) were transferred into new MSM solutions with corresponding hydrocarbons every ten days for 40 d. Residuals were kept at −80 °C for bacterial community analysis. 40 dAll the treatments at the first two transformations were performed in duplicate. For accurate chemical analysis, quadruplicate treatments were setup for each hydrocarbon at the last transformation. Two of the quadruplicating samples were specially used for chemical analysis, in case of any loss of hydrocarbons in solutions or on flasks. And the other two were applied for analysis of bacterial communities at the day 40. Controls were performed by adding 400 mg/L of different hydrocarbons in MSM medium without inoculation of sediment.

### Extraction and quantification of hydrocarbons

Hydrocarbons in liquid solutions were dissolved in dichloromethane, and all solutions were transferred into separatory funnel. Flasks were then washed twice by 20 ml of dichloromethane and then mixed with precious solutions. Hydrocarbons were then extracted using dichloromethane three times and all organic phase was collected. Dehydration of collected solution was performed by addition of excessive magnesium sulfate (MgSO_4_). Dehydrated solutions were then concentrated by using rotary evaporator to a final volume of 10 ml. Extracted hydrocarbons were analyzed by GC-MS (Agilent 7890-5975c) equipped with a DB-5 capillary column (30 m × 250 μm × 0.25 μm, Agilent Technologies, U.S.A.). High purity helium (99.999%) was applied as the carrier gas with the speed of 1 ml/min. The temperature program was conducted as follows, 50–200 °C at 25 °C/min, 200–260 °C at 15 °C/min, and 260 °C for 10 min. Injector and transfer line temperatures were both set at 260 °C. Degradation efficiency was calculated by comparing peak areas of hydrocarbons.

### DNA extraction and Miseq sequencing

Total genomic DNA was extracted by using PowerSoil^®^ DNA Isolation Kit (MoBio Laboratories, Solana Beach, CA). DNA concentration and quality were determined by using a NanoDrop 2000 UV-Vis Spectrophotometer (Thermo Scientific, Wilmington, DE). High quality DNA was then applied for Miseq sequencing for amplifying V4 hypervariable region of 16S rRNA gene. The primer set used in this study was universal primer 515F (5′-GTGCCAGCMGCCGCGGTAA-3′) and 806R (5′-GGACTACHVGGGTWTCTAAT-3′) with 12 nt unique barcode. The sequence data were analyzed by using QIIME Pipeline–Version 1.9.0 (http://qiime.org/). After being trimmed and assigned to each sample based on barcodes, sequences with high quality (length > 150 bp, without ambiguous base ‘N’, and average base quality score >30) were chimera checked by using usearch quality filter (usearch_qf)^50^,^51^. Sequences were clustered into operational taxonomic units (OTUs) at a 97% identity threshold by using the Uclust algorithm^51^. To standardize sequencing data from different samples, sequences were normalized to 8000 sequences using random screening as described by Fortunato *et al*.^52^. Remaining OTUs were identified and assigned to different taxonomy using RDP (Ribosomal Database Project) classifier retained with Greengenes database^53^. The sum of sequences of each OTU in all 42 samples was calculated and OTUs containing less than 5 sequences were not considered for further analysis.

### Statistical test for bacterial communities after domestication

Network profiles were constructed to verify the stationary of bacterial communities as follows. Bacterial phylotypes showing significant differences (F-test < 0.1) were used to construct networks by applying the open source platform, Cytoscape^54^. CytoCluster plugin was applied to further cluster the generated network.

### Analysis of successions of bacterial communities

Successions of bacterial communities of the whole domesticating period were analyzed based on 16S rRNA gene sequencing data and assigned taxonomic information. First, all sequences were classified to different bacterial phyla and different classes in the phylum proteobacteria. Relative abundances of different bacterial phylotypes at phylum-level (class level in proteobacteria) were then calculated and visualized by using ggplot2 packages in R^55^. Bacterial phylotypes with significant differences after 40 d domestication (F-test < 0.1) were applied for drawing heatmap profile and constructing network to analyze the succession of bacterial communities and their responses to stresses of different hydrocarbons at the genus-level. Heatmap profile was generated by using HemI-1.0.1-Heatmap Illustrator (http://hemi.biocuckoo.org/faq.php), while network was constructed by using cytoscape. Bacterial phylotypes showing dramatic changes (F-test < 0.1) where selected as source interaction elements, and hydrocarbons were regarded as target interaction elements when network was constructed. Interactions between source and target elements were conducted based on different types of responses of bacterial communities to stresses of varied hydrocarbons (increase, decrease and disappear).

### Function prediction and metabolic pathway reconstruction

PICRUSt (Phylogenetic Investigation of Communities by Reconstruction of Unobserved States^39^, and KEEG (Kyoto Encyclopedia of Genes and Genomes)^38^ were applied for predicting functional responses of individual bacterial taxa in special bacterial communities to different hydrocarbons based on 16S rRNA sequencing data. OTUs harboring genes involved in the reactions of degrading alkanes and PAHs were predicted using the green gene numbers of OTUs obtained and PICRUSt. Contribution of these OTUs to the given genes was automatically estimated by using the KEGG Orthology database.

### Network construction and function analysis

To investigate the co-acclimation of bacterial communities and define functions of different bacterial groups, networks were constructed for bacterial communities with bacterial phylotypes, showing significant correlation evaluated by Pearson’s correlation analysis (*p* < 0.05, |r| > 0.8). Networks were also generated by Cytoscape as mentioned above and split into different functional groups (subnetworks) by using “Cytocluster” in the module. Functions were endowed to different sub-networks based on predicted, functions of dominant bacterial phylotypes in the group.

## Data Availability

Raw sequencing data with BioSample accession No. SAMN04589160-SAMN04589201 were available at Sequence Read Archive at the website of National Center for Biotechnology Information (NCBI).

## Additional Information

**How to cite this article**: Wang, H. *et al*. Co-acclimation of bacterial communities under stresses of hydrocarbons with different structures. *Sci. Rep.* **6**, 34588; doi: 10.1038/srep34588 (2016).

## Supplementary Material

Supplementary Information
