# Supplementary material for: Co-acclimation of bacterial communities under stresses of hydrocarbons with different structures
Source: Sci Rep. 2016 Oct 4;6:34588. doi: 10.1038/srep34588 (PMC5048299; doi:10.1038/srep34588)
Supplement: Supplementary Information [file srep34588-s1.pdf]

Co-acclimation of bacterial communities under stresses of hydrocarbons with different structures

Hui Wang[$], Bin Wang[$], Wenwen Dong, Xiaoke Hu*

Key Laboratory of Coastal Biology and Bioresource Utilization, Yantai Institute of Costal Zone Research, Chinese Academy of Sciences, Yantai, China 264003;

Running title: Co-acclimation of bacteria

*Corresponding author. E-mail: xkhu@yic.ac.cn (XH).

[$]These authors contributed equally to this work.

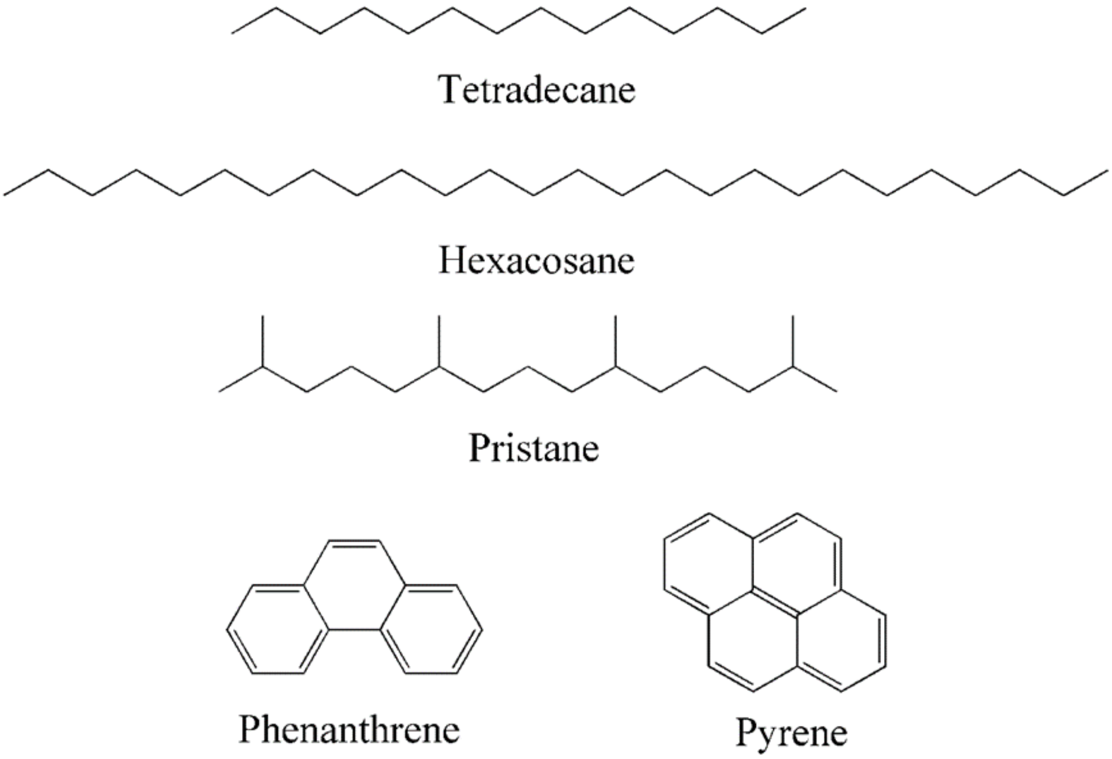

Supplemental Figure S1. Hydrocarbons with different structures that are used in this study.

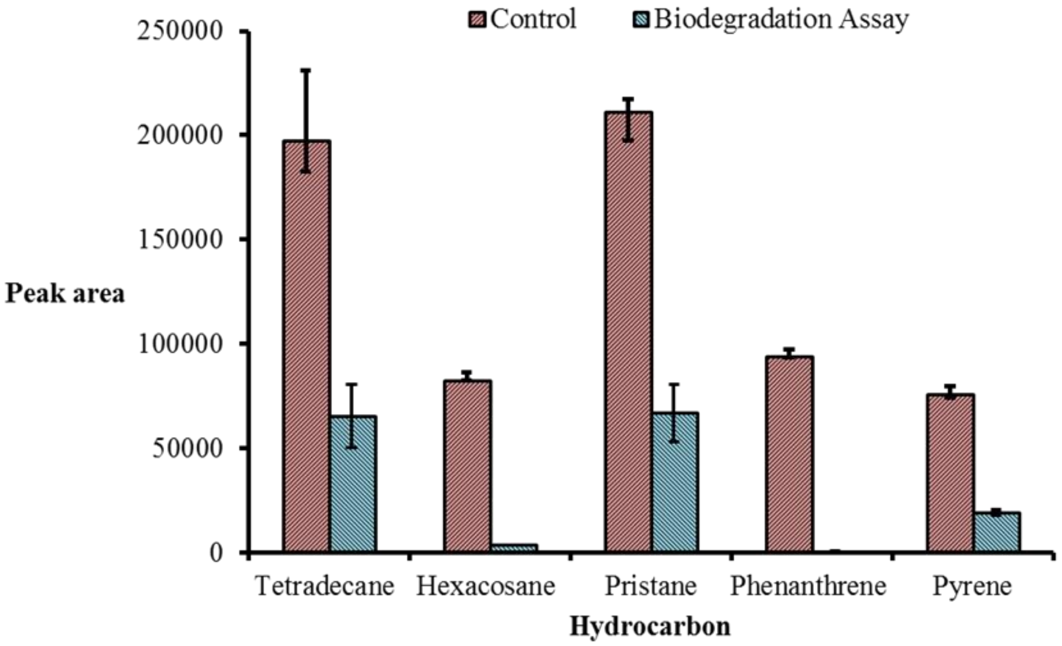

Supplemental Figure S2. Biodegradation of different hydrocarbon at the last domesticating period (form the 30 days to 40 days). Peak areas indicating remaining hydrocarbons in “control” and “Biodegradation Assay” were measured by GC-MS. All measurements were conducted in duplicates.

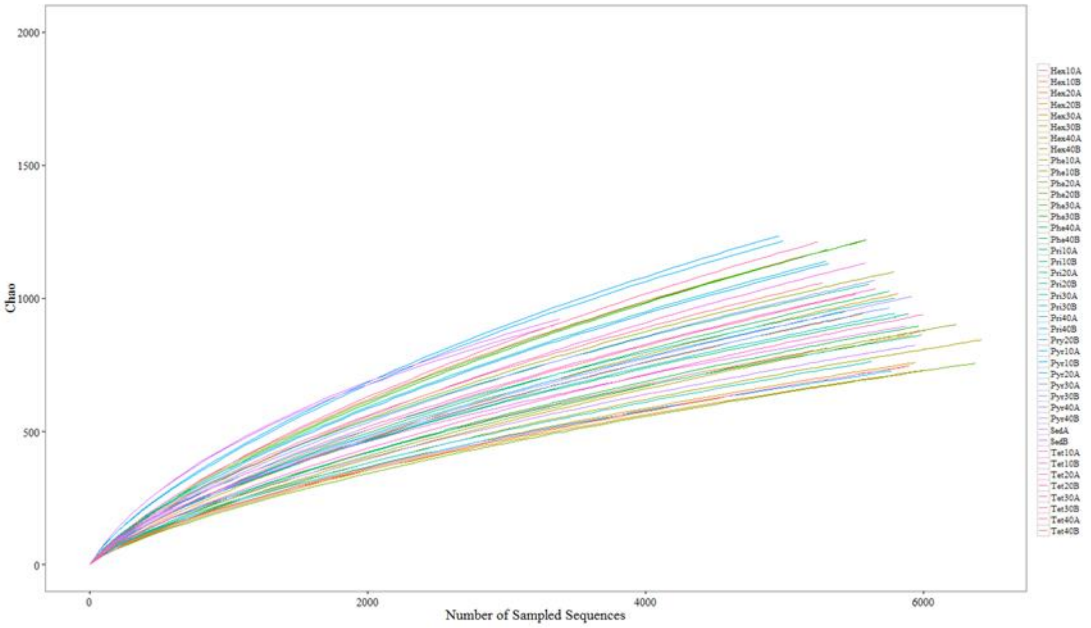

Supplemental Figure S3. Rarefaction curve generated from the observed species for all the sequenced samples. Tet--Tetradecane, Hex--Hexacosane, Pri--Pristane, Phe--Phenanthrene, Pyr--Pyrene, Sed—Original sediments. 1 and 2 were samples collected at 10 days, 3 and 4 were samples collected at 20 days, 5 and 6 were samples collected at 30 days, 7 and 8 were samples collected at 40 days.

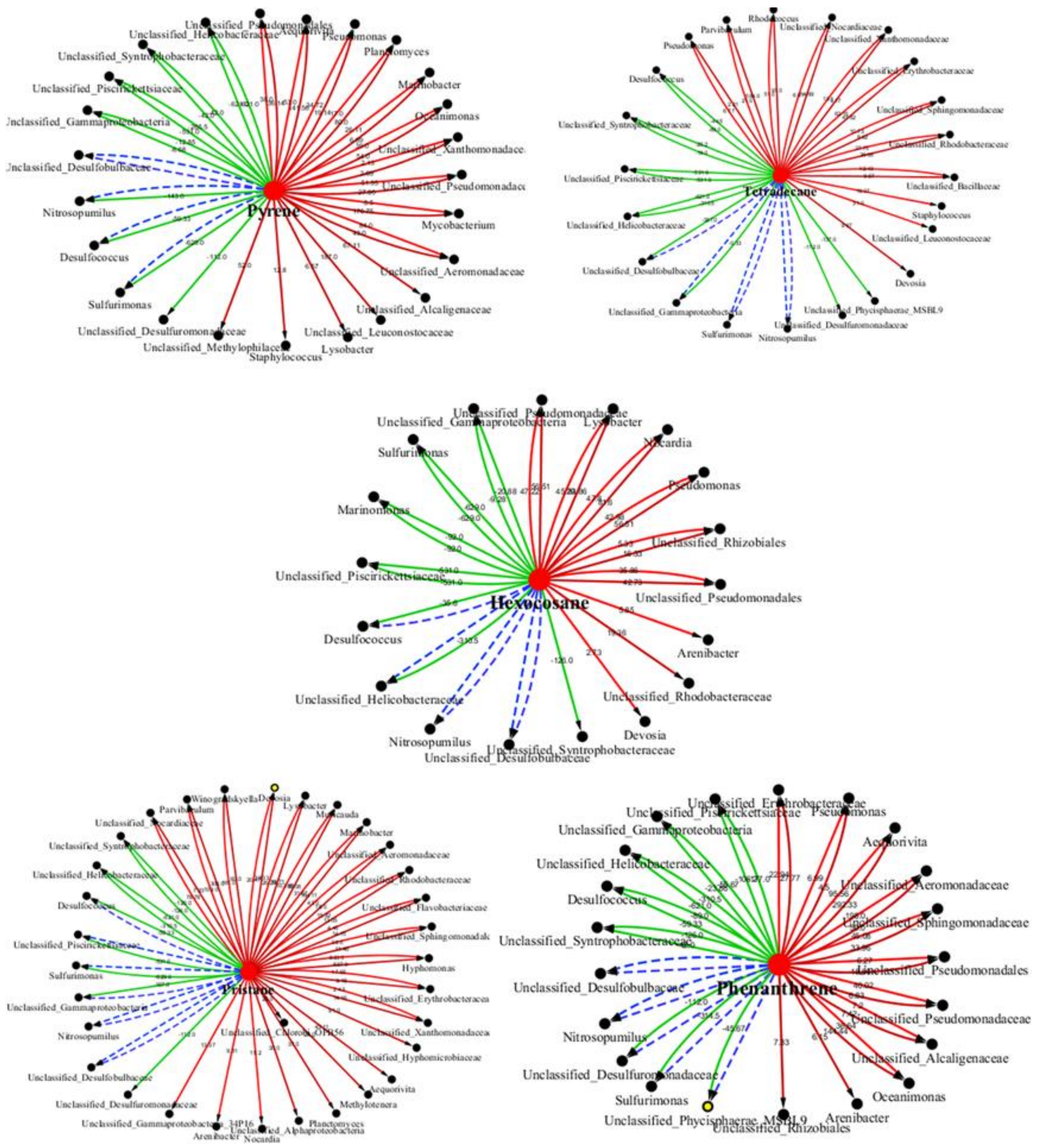

Supplemental Figure S4. Network profiling describing the stationary of bacterial communities, after co-incubation with different hydrocarbons. Red dots represent different hydrocarbons, while black dots represent bacterial phylotypes. Which shows significant differences (F-test<0.09) between the number of bacteria found in sediment after 30 d or 40 d old samples. Blue dash lines indicated that the phylotype did not existed after 30 d or 40 d after demonstration (could use experiment if you want). Red solid lines indicated sequencing number that increased at 30 d or 40 d old samples. Green solid lines indicated sequencing number decreased at 30 d or 40 d old samples.

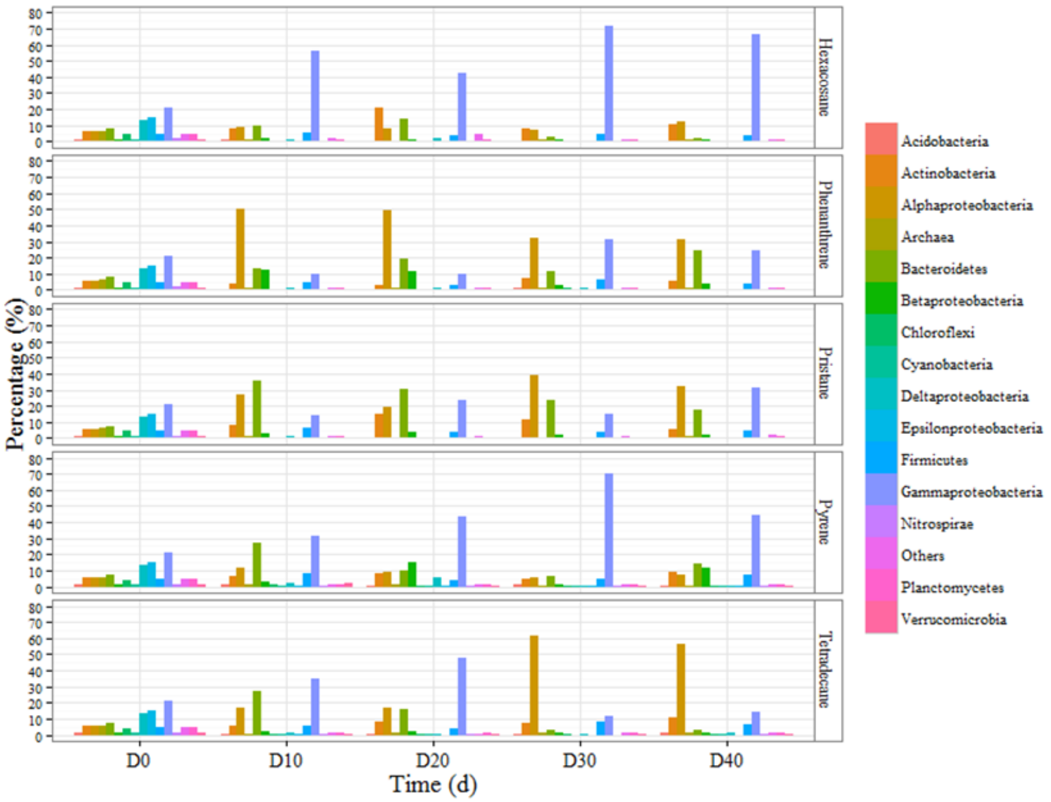

Supplemental Figure S5. Relative abundance of different bacterial phyla and Proteobacteria classes, when treated with five different hydrocarbons for 40 days. ‘Other’ included all the sequences that lower than 1% of total OTUS. MiSeq data were generated from duplicate samples.

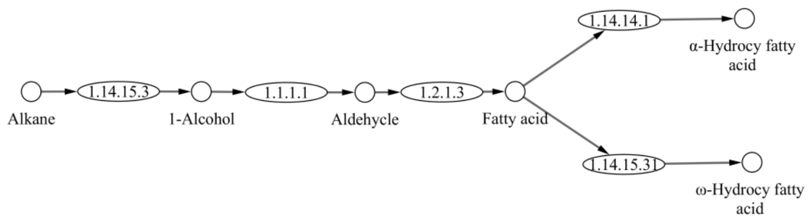

Supplemental Figure S6. The predicted pathway of alkane degradation modified from KEEG. Circles, ellipse, enzymes catalyzing, alkane and metabolite; for each reaction.

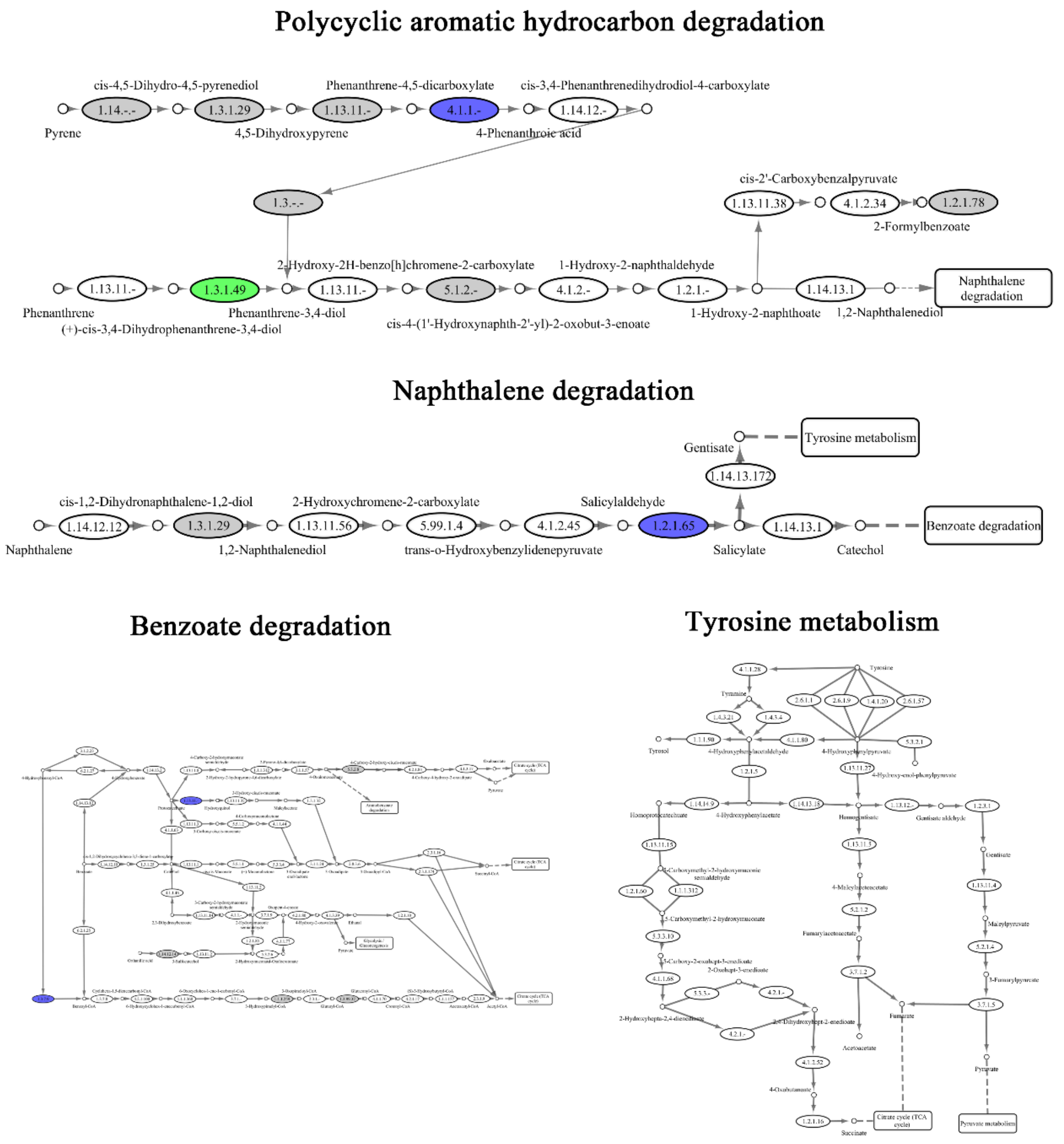

Supplemental Figure S7. The Predicted pathway of pyrene and phenathrene degradation modified from KEGG. Circles, pyrene/phenathrene and their metabolite; ellipse, enzymes catalyzing for each reaction. Hollow ellipse meant functional genes related the enzyme were detected from bacterial communities of both pyrene and phenathrene treatments. Grey ellipse meant functional genes related the enzyme was not detected from bacterial communities of both treatments. Blue ellipse meant functional genes related the enzyme was only detected from bacterial communities of phenathrene treatment. Green ellipse meant functional genes related the enzyme was only detected from bacterial communities of pyrene treatment.

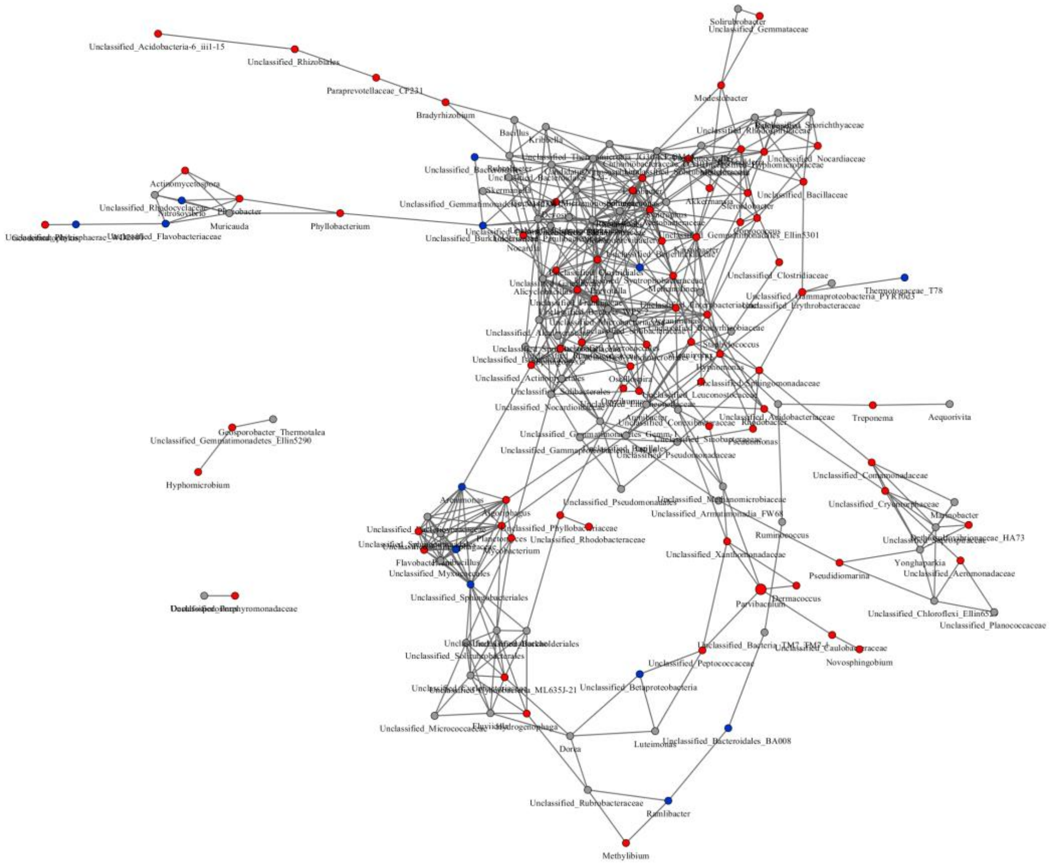

Supplemental Figure S8. Network profiling showing interactions between different bacterial phylotypes with the stress of tetradecane. Red and blue dots represent increased and decreased bacterial phylotypes after 40 days co-incubation, respectively. Grey dots represented bacterial phylotypes without dramatic changes (0.5<Sequencing number of 40 d/sediment<2) after 40 days.

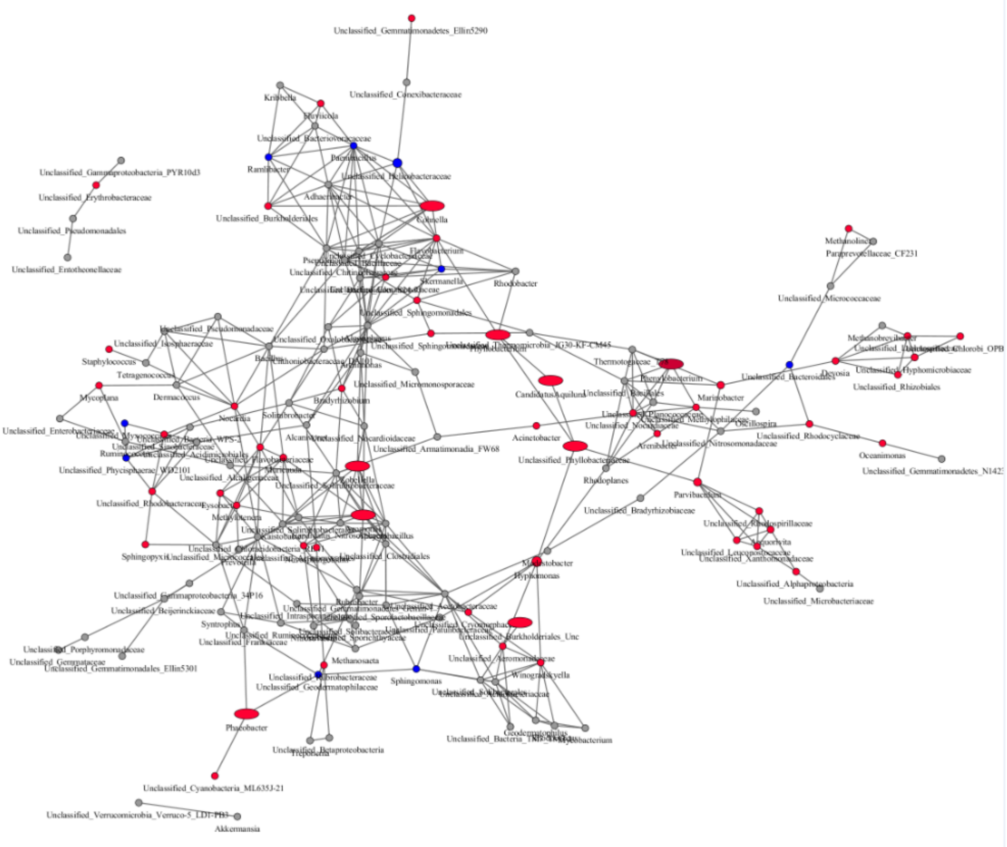

Supplemental Figure S9. Network profiling showing interactions between different bacterial phylotypes with the stress of pristane. Red and blue dots represent increased and decreased bacterial phylotypes after 40 days co-incubation, respectively. Grey dots represent bacterial phylotypes without dramatic changes (0.5<Sequencing number of 40 d/sediment<2) after 40 days.

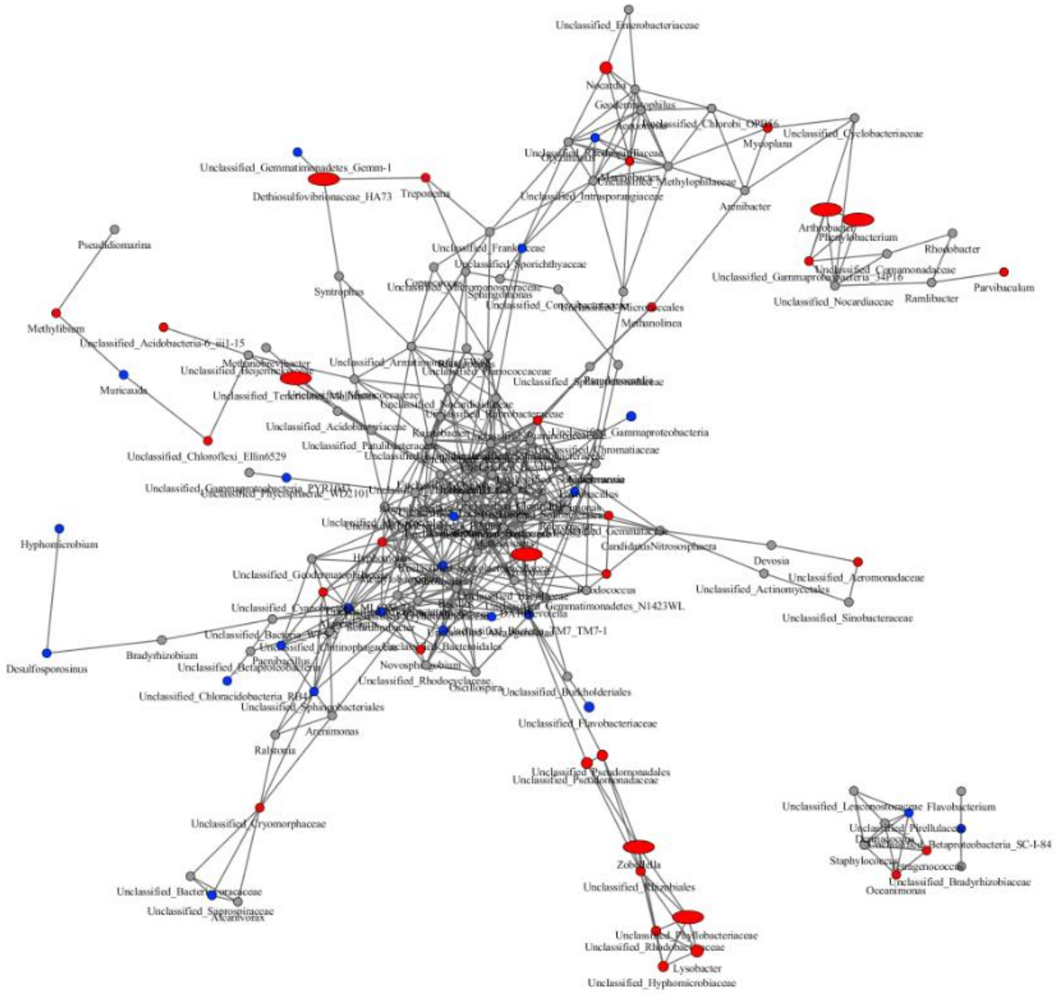

Supplemental Figure S10. Network profiling showing interactions between different bacterial phylotypes with the stress of hexacosane. Red and blue dots represent increased and decreased bacterial phylotypes after 40 days co-incubation, respectively. Grey dots represent bacterial phylotypes without dramatic changes (0.5<Sequencing number of 40 d/sediment<2) after 40 days.

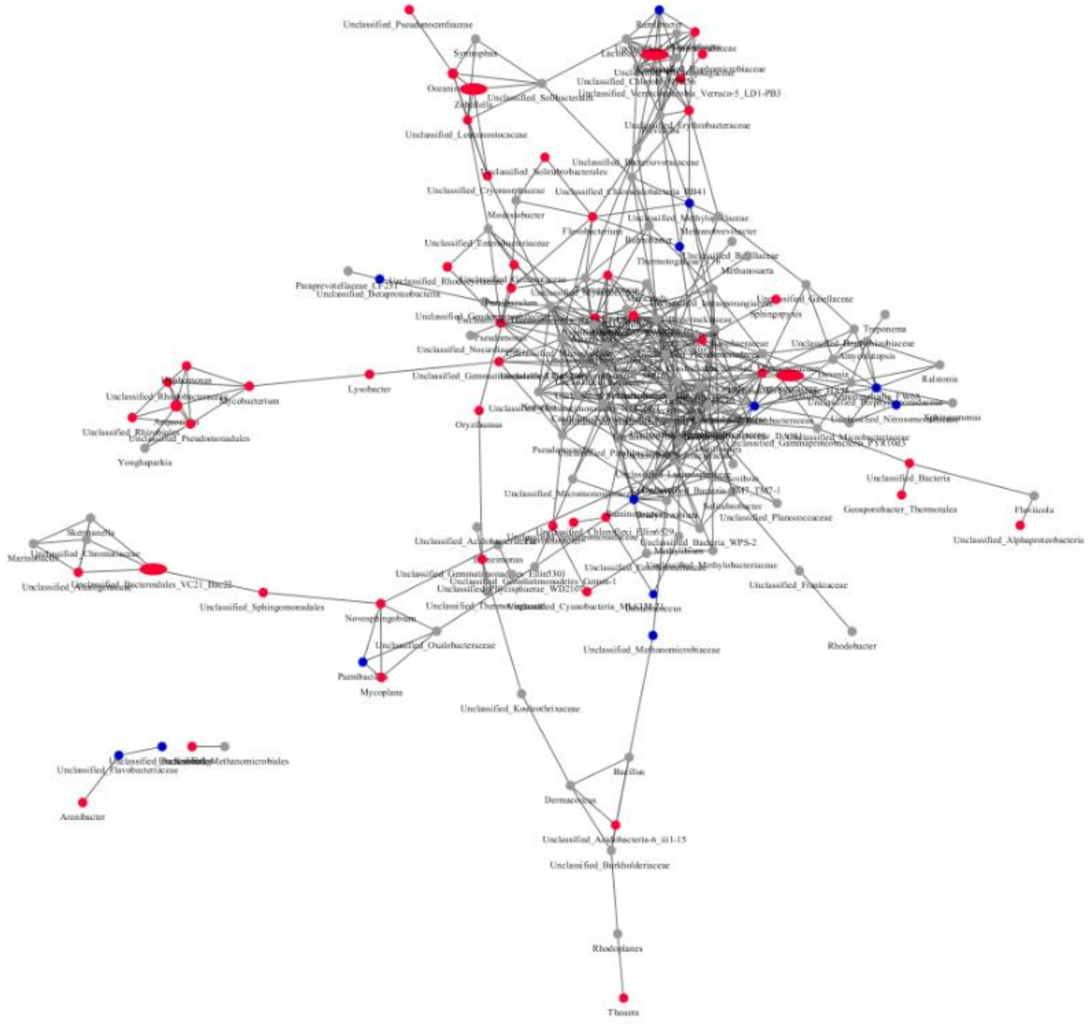

Supplemental Figure S11. Network profiling showing interactions between different bacterial phylotypes with the stress of phenathrene. Red and blue dots represent increased and decreased bacterial phylotypes after 40 days co-incubation, respectively. Grey dots represent bacterial phylotypes without dramatic changes (0.5<Sequencing number of 40 d/sediment<2) after 40 days.

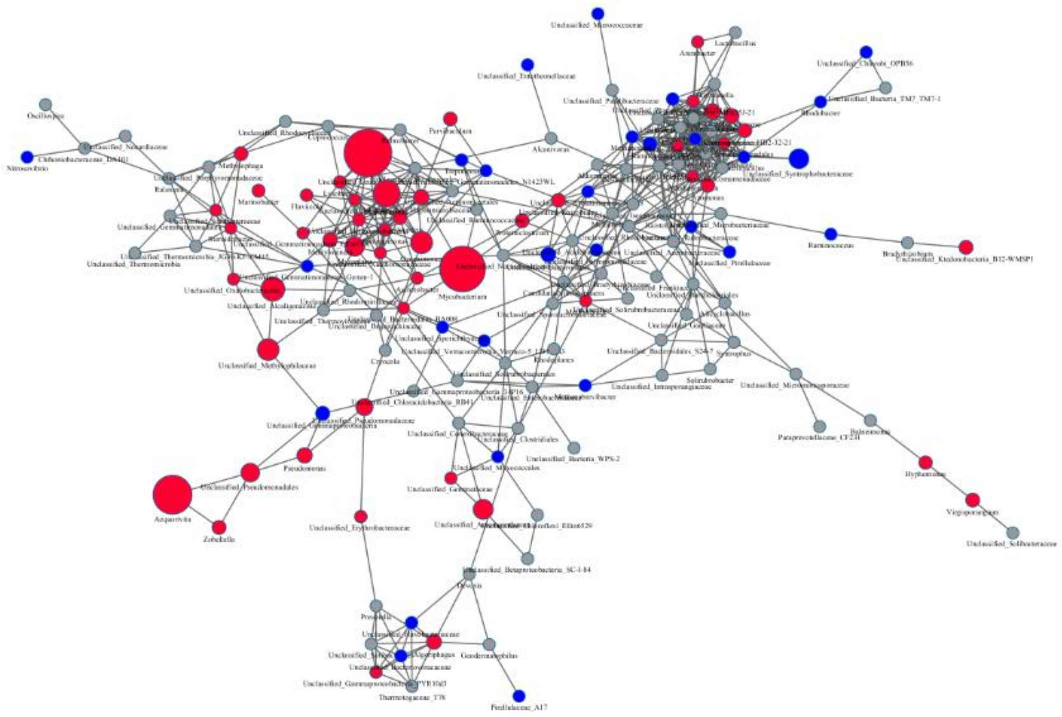

Supplemental Figure S12. Network profiling showing interactions between different bacterial phylotypes with the stress of pyrene. Red and blue dots represent increased and decreased bacterial phylotypes after 40 days co-incubation, respectively. Grey dots represent bacterial phylotypes without dramatic changes (0.5<Sequencing number of 40 d/sediment<2) after 40 days.

Supplemental Table S1. Predicted functions of sub-networks; composed from the network profile of tetradecane EC No. in the table indicates the number of predicated enzymes. The bacterial phylotypes indicated may involve in the biochemical pathways and the tetradecane degradation pathway. Bold enzyme numbers indicate that the functional bacterial group contributed greater than (>10%) to the encoded gene of the enzyme.

Clusters	Sub-networks	Functional bacteria	Enzyme Numbers (EC No.)	Involved steps
**1**	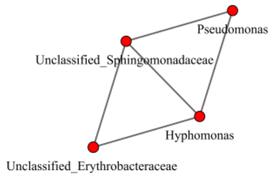	Pseudomonas	**1.1.1.1, 1.14.15.3,** 1.2.1.3	**1**, **2**, 3, **4**
		Unclassified Sphingomonadaceae	1.2.1.3, 1.14.14.1, 1.1.1.1	2, 3, 4
		Unclassified Erythrobacteraceae	**1.14.14.1, 1.1.1.1, 1.2.1.3**	**2, 3, 4**
		Hyphomonas Hyphomonas	1.2.1.3, 1.1.1.1	2, 3
**2**	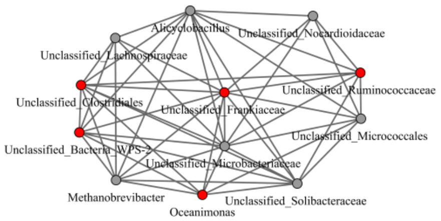	Unclassified Solibacteraceae	**1.1.1.1**, 1.2.1.3	2, 3
		Oceanimonas Unclassified	1.14.15.3, 1.1.1.1, 1.2.1.3	1, 2, 3, 4
		Unclassified Ruminococcaceae	1.1.1.1, 1.2.1.3	2, 3
		Unclassified Microbacteriaceae	1.1.1.1, 1.2.1.3	2, 3

	Unclassified Micrococcales	1.1.1.1, 1.2.1.3	2, 3
	Unclassified Solibacteraceae	1.1.1.1, 1.2.1.3	2, 3
	Oceanimonas	1.1.1.1, 1.2.1.3	2, 3
	Unclassified Clostridiales	1.1.1.1, 1.2.1.3	2, 3
	Unclassified Frankiaceae	1.1.1.1, 1.2.1.3	2, 3
	Unclassified Nocardioidaceae	1.1.1.1, 1.14.14.1, 1.14.15.3, 1.2.1.3	1, 2, 3, 4
	Alicyclobacillus	1.1.1.1, 1.2.1.3	2, 3

Supplemental Table S2. Predicted functions of sub-networks; composed from the network profile of pristane. EC No. in the table indicates the number of predicated enzymes. The bacterial phylotypes indicated may involve in the biochemical pathways and the pristane degradation pathway. Bold enzyme numbers indicate that the functional bacterial group contributed greater than (>10%) to the encoded gene of the enzyme.

**Clusters**	**Sub-networks**	**Functional bacteria**	**Enzyme Numbers (EC No.)**	**Involved steps**
**1**	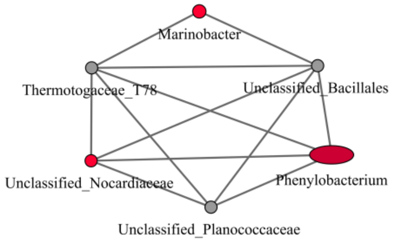	Marinobacter	**1.14.15.3,** 1.1.1.1, 1.2.1.3	**1**, 2, 3, **4**
		Unclassified Bacillales	1.2.1.3, 1.1.1.1	2, 3
		Unclassified Nocardiaceae	**1.14.14.1, 1.14.15.3, 1.1.1.1, 1.2.1.3**	**1, 2, 3, 4**
**2**	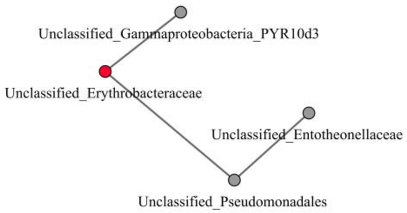	Unclassified Erythrobacteraceae	**1.14.14.1, 1.2.1.3,** 1.1.1.1	2, **3, 4**
		Unclassified_Pseudo monadales	1.14.15.3, 1.1.1.1, 1.2.1.3	1, 2, 3, 4

**3**	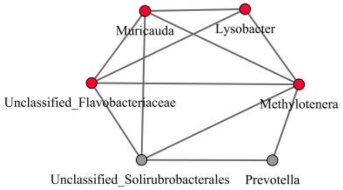	Muricauda	**1.14.15.3, 1.2.1.3**	**1, 3, 4**
		Lysobacter	1.2.1.3, 1.1.1.1	2, 3
		Methylotenera	1.2.1.3	3
		Unclassified Flavobacteriaceae	1.2.1.3, 1.14.15.3, 1.1.1.1	**1, 2, 3, 4**

Supplemental Table S3. Predicted functions of sub-networks decomposed from network profile of hexacosane. EC No. in the table indicated enzymes predicated from the bacterial phylotypes and involved pathways represented pathways the phylotypes might involve in the hexacosane degradation pathway. Bold enzyme numbers indicated that the functional bacterial group contributed great (>10%) to the encoded gene of the enzyme.

**Clusters**	**Sub-networks**	**Functional bacteria**	**Enzyme Numbers (EC No.)**	**Involved steps**
**1**	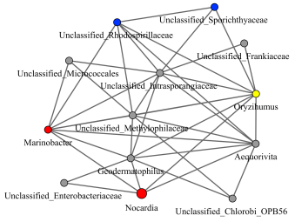	Nocardia	**1.14.15.3, 1.14.14.1, 1.2.1.3, 1.1.1.1**	**1, 2, 3, 4**
		Aequorivita	1.2.1.3	3
		Unclassified Frankiaceae	1.1.1.1, 1.2.1.3	2, 3
		Unclassified Micrococcales	1.1.1.1, 1.2.1.3	2, 3
		Unclassified Intrasporangiaceae	1.1.1.1, 1.2.1.3	2, 3
**2**	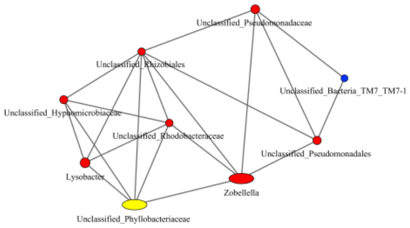	Lysobacter	1.1.1.1, 1.2.1.3	2, 3
		Unclassified Hyphomicrobiaceae	1.1.1.1, 1.2.1.3	2, 3
		Unclassified Rhizobiales	1.14.14.1, 1.2.1.3, 1.1.1.1	2, 3,4
		Unclassified Rhodobacteraceae	1.14.15.3, 1.14.14.1, 1.2.1.3, 1.1.1.1	1, 2, 3, 4
		Unclassified Pseudomonadales	**1.14.15.3**, 1.2.1.3, 1.1.1.1	**1**, 2, 3, **4**
		Unclassified Pseudomonadaceae	**1.14.15.3**, 1.2.1.3, 1.1.1.1	**1**, 2, 3, **4**

Supplemental Table S4. Predicted functions of sub-networks; composed from the network profile of phenanthrene. EC No. in the table indicates the number of predicated enzymes. The bacterial phylotypes indicated may involve in the biochemical pathways and phenanthrene degradation pathway. Bold enzyme numbers indicate that the functional bacterial group contributed greater than (>10%) to the encoded gene of the enzyme.

Clusters	Sub-networks	Functional bacteria	Enzyme Numbers (EC No.)	Involved pathways
**1**	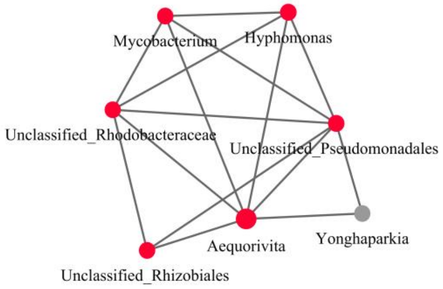	Mycobacterium	**1.13.11.-, 1.13.11.38, 4.1.2.-, 1.2.1.-, 4.1.2.34, 1.4.3.21,** 1.2.1.10, 1.13.11.37, 1.14.13.1, 1.4.3.4, 4.1.1.44, 5.5.1.2, 4.2.1.17, 2.3.1.-, 2.3.1.9, 4.1.3.39, 4.1.1.77, 2.3.1.16, 2.8.3.6, 1.13.11.5, 2.6.1.9, 4.2.1.80, 1.1.1.157, 4.1.1.46, 1.2.1.16, 5.3.3.4, 1.13.11.3, 1.14.12.-, 1.14.14.9, 1.13.11.1, 1.1.1.90, 3.1.1.24, 1.2.1.60, 1.14.12.10, 1.13.11.4, 3.1.2.23, 1.13.11.2, 5.3.2.6, 1.14.12.10	PAH and Tyrosine degradation
		Hyphomonas	1.13.11.4, 1.14.13.1, 2.6.1.1, 5.3.3.10, 4.1.1.44, 5.2.1.2, 2.3.1.-, 4.2.1.83, 1.1.1.90, 1.13.11.8, 4.2.1.80, 5.3.2.6, 4.2.1.17, 2.6.1.9, 2.3.1.9, 1.2.1.16, 1.13.11.27, 1.1.1.157, 2.6.1.1	NA
		Unclassified Rhodobacteraceae	**6.2.1.27,** 4.1.1.28, 4.1.3.17, 1.1.1.312, 1.14.13.1, 3.1.1.57, 1.13.11.15, 1.14.13.2, 1.13.11.3, 5.5.1.2, 4.1.1.44, 3.1.1.24, 1.14.12.-, 5.3.3.10, 1.4.3.4, 1.13.11.4, 3.1.2.23, 1.13.11.2, 3.7.1.2, 1.2.1.60, 2.3.1.-, 1.13.11.5, 2.6.1.9, 2.6.1.57, 1.2.1.16, 4.2.1.17, 2.3.1.9, 1.13.11.27, 5.2.1.2, 2.6.1.1, 1.13.11.8, 1.1.1.157, 5.3.2.6, 1.13.11.2, 1.13.11.1, 4.1.3.39, 4.1.1.46, 2.3.1.16, 4.2.1.80, 5.5.1.1	Benzoate degradation

		Unclassified Pseudomonadales	**3.1.2.23, 4.1.1.44, 3.1.1.24,** 1.13.11.5, 2.6.1.9, 5.2.1.2, 2.6.1.57, 2.3.1.9, 1.2.1.16, 2.3.1.-, 1.13.11.27, 4.2.1.17, 1.1.1.157, 2.6.1.1, 2.3.1.16	Benzoate degradation
		Aequorivita	**1.2.1.60, 2.3.1.-, 3.7.1.2, 1.13.11.5, 1.1.1.157, 2.6.1.1, 1.13.11.27, 2.6.1.1, 2.6.1.9, 2**.3.1.16, 1.2.1.16, 2.3.1.9, 4.2.1.17	Benzoat and Tyrosine degradation
		Unclassified Rhizobiales	**1.2.1.65, 1.3.1.32, 4.1.1.28, 4.1.2.45,** 3.1.1.57, 1.13.11.15, 4.1.3.17, 1.14.13.2, 1.14.13.1, 1.13.11.3, 1.13.11.1, 5.5.1.2, 3.1.1.24, 1.13.11.2, 1.13.11.2, 4.1.1.44, 1.4.3.4, 3.1.2.23, 1.13.11.4, 1.2.1.10, 2.3.1.-, 1.4.3.21, 5.3.3.10, 5.2.1.2, 5.3.2.6, 1.2.1.16, 1.13.11.5, 2.6.1.1, 2.6.1.9, 1.2.1.60, 1.13.11.27, 4.2.1.17, 2.3.1.9, 4.1.1.46, 1.1.1.157, 5.5.1.1, 2.3.1.16, 4.1.1.77, 2.8.3.6, 2.6.1.57, 5.99.1.4, 4.1.3.39, 3.7.1.5	Naphthalene, Tyrosine and Benzoate degradation
**2**	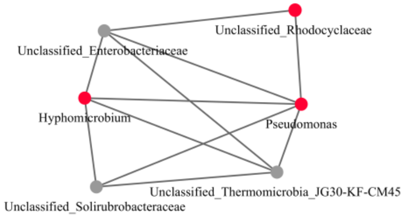	Unclassified Rhodocyclaceae	**3.7.1.-, 5.2.1.4, 1.1.1.368, 4.2.1.100, 6.2.1.25, 1.2.1.85, 1.14.12.-, 1.3.7.9, 1.2.1.10, 3.1.2.23, 1.13.11.2, 1.14.13.1, 1.13.11.4,** 4.2.1.80, 4.1.3.39, 2.3.1.-, 4.1.1.44, 1.1.1.157, 1.13.11.8, 2.3.1.16, 4.1.1.77, 5.3.2.6, 2.6.1.57, 2.6.1.9, 5.2.1.2, 4.2.1.17, 2.3.1.9, 1.2.1.16, 1.1.1.90	Naphthalene, Tyrosine and Benzoate degradation
		Pseudomonas	**1.13.11.15, 4.1.1.68, 1.14.14.9, 2.3.1.174, 5.3.3.10, 1.13.11.3, 1.14.14.9, 5.5.1.2, 1.14.13.2, 1.3.1.25, 3.1.2.23, 4.1.1.44, 5.3.3.4, 3.1.1.24, 1.13.11.1,** 1.4.3.4, 1.14.12.10, 1.14.12.10, 2.6.1.57, 1.2.1.60, 3.7.1.2, 1.13.11.5, 1.13.11.27, 1.2.1.10, 2.6.1.9, 5.2.1.2, 2.3.1.9, 1.1.1.157, 2.3.1.16, 4.2.1.17, 5.3.2.6, 5.5.1.1, 1.2.1.16, 2.3.1.-, 2.6.1.1, 4.1.3.39, 1.14.12.10, 4.2.1.80, 1.14.12.-, 1.14.13.1	Tyrosine and Benzoate degradation

		Unclassified Solirubrobacteraceae	1.13.11.4, 1.13.11.1, 1.2.1.10, 1.13.11.2, 1.4.3.4, 3.1.1.24, 1.1.1.157, 4.2.1.17, 1.13.11.5, 1.1.1.90, 4.1.1.77, 4.1.3.39, 1.2.1.16, 2.3.1.9, 2.6.1.9, 2.3.1.16	NA
**3**	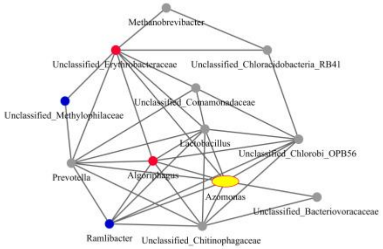	Unclassified Erythrobacteraceae	**4.1.1.46, 4.2.1.83, 5.99.1.4, 1.1.1.90, 1.13.11.8, 4.2.1.80, 5.5.1.1, 4.1.1.77, 4.1.3.39, 5.3.2.6, 4.2.1.17, 5.2.1.2, 2.6.1.57, 2.3.1.9, 2.3.1.16, 1.2.1.16, 1.13.11.27, 1.1.1.157, 2.6.1.9, 1.14.12.12, 1.14.12.10, 4.1.2.45, 1.13.11.56, 1.1.1.312, 3.1.1.57, 1.2.1.85, 4.1.3.17, 1.3.1.25, 1.2.1.10, 1.13.11.2, 1.13.11.4, 1.14.12.10,** 1.14.13.2, 4.1.1.68, 3.7.1.2, 1.2.1.60, 2.6.1.1, 2.3.1.-	Tyrosine and Benzoate degradation
		Unclassified Comamonadaceae	**4.1.2.34, 1.14.12.12, 1.13.11.37, 1.1.1.312, 1.14.12.12, 3.1.1.57, 1.3.1.32, 4.1.3.17,** 2.8.3.6, 6.2.1.25, 1.2.1.85, 1.14.13.1, 1.14.12.-, 1.14.14.9, 4.1.1.68, 1.2.1.10, 5.5.1.2, 1.13.11.4, 5.3.3.10, 1.14.13.2, 3.1.2.23, 1.13.11.2, 4.1.1.44, 4.1.1.77, 3.1.1.24, 3.7.1.2, 5.3.2.6, 1.2.1.60, 4.1.2.45, 2.6.1.57, 5.3.3.4, 2.6.1.9, 1.1.1.157, 4.2.1.17, 2.3.1.9, 1.13.11.8, 4.1.3.39, 4.2.1.83, 5.2.1.2, 1.4.3.4, 1.13.11.3, 2.3.1.-, 1.13.11.27, 2.3.1.16, 1.2.1.16, 4.2.1.80, 1.13.11.5, 1.13.11.1, 1.14.12.10, 2.6.1.1, 1.3.1.25, 1.14.12.10, 5.5.1.1, 4.1.1.46, 5.99.1.4, 1.14.12.12, 1.14.12.10	PAH, Naphthalene, Benzoate and Tyrosine degradation

	Prevotella	2.6.1.1, 2.3.1.-, 4.1.1.44, 2.6.1.1, 2.6.1.9	NA
	Unclassified Chitinophagaceae	**1.4.3.4,** 3.7.1.2, 1.13.11.5, 1.1.1.157, 2.6.1.9, 2.6.1.1, 1.2.1.16, 1.13.11.27, 2.6.1.1, 2.3.1.16, 2.3.1.9, 1.13.11.2, 4.2.1.17	Tyrosine degradation
	Unclassified Bacteriovoracaceae	4.1.1.44, 2.3.1.-, 1.13.11.5, 1.1.1.157, 1.13.11.27, 2.3.1.9, 2.6.1.1, 2.6.1.9, 2.3.1.16, 4.2.1.17, 1.2.1.16	NA
	Lactobacillus	4.1.1.44, 2.3.1.-, 5.3.2.6, 5.5.1.2, 1.2.1.16, 1.1.1.90, 1.13.11.2, 1.1.1.157, 2.3.1.9, 2.6.1.9	NA

Supplemental Table S5. Predicted functions of sub-networks decomposed from network profile of pyrene. EC No. in the table indicated enzymes predicated from the bacterial phylotypes and involved pathways represented pathways the phylotypes might involve in the pyrene degradation pathway. Bold enzyme numbers indicated that the functional bacterial group contributed great (>10%) to the encoded gene of the enzyme.

Clusters	Sub-networks	Functional bacteria	Enzyme Numbers (EC No.)	Involved pathways
1	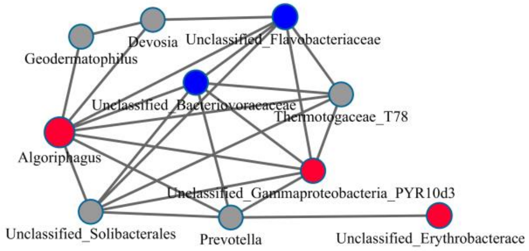	Unclassified Erythrobacteraceae	**5.99.1.4, 1.14.12.12, 4.2.1.83, 1.13.11.8, 1.1.1.90, 4.1.1.46, 1.14.12.10, 5.5.1.1, 4.2.1.80, 4.1.2.45, 5.3.2.6, 4.1.1.77, 4.1.3.39, 5.2.1.2, 2.6.1.57,** 1.2.1.16, 2.3.1.16, 1.13.11.27, 4.2.1.17, 2.3.1.9, 1.1.1.157, 2.6.1.9, 1.13.11.2, 1.13.11.4, 1.3.1.25, 1.14.12.10, 3.7.1.2, 1.2.1.10, 2.6.1.1, 3.1.1.24, 4.1.1.44	Naphthalene, Tyrosine and Benzoate degradation
		Unclassified Gammaproteobacteria PYR10d3	2.6.1.1, 2.6.1.9, 1.2.1.16, 5.3.2.6, 2.3.1.9, 2.3.1.16, 4.1.1.44, 4.2.1.17	NA
		Unclassified Flavobacteriaceae	2.6.1.1, 1.2.1.60, 3.7.1.2, 2.6.1.1, 2.3.1.-, 1.13.11.5, 1.13.11.27, 1.1.1.157, 5.3.3.10, 1.2.1.16, 2.3.1.16, 2.6.1.9, 1.4.3.4, 2.3.1.9, 4.2.1.17, 1.13.11.2	NA
2	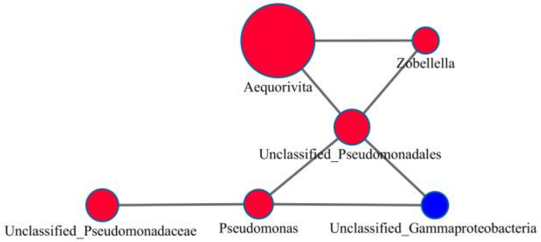	Aequorivita	**2.6.1.1, 1.2.1.60, 3.7.1.2,** 2.3.1.-, 1.1.1.157, 2.6.1.1, 1.13.11.5, 1.13.11.27, 1.2.1.16, 2.3.1.16, 2.6.1.9, 2.3.1.9, 4.2.1.17	Tyrosine degradation
		Zobellella	3.7.1.5, 2.6.1.1, 1.13.11.4, 1.14.12.10, 1.3.1.25, 1.14.12.10, 5.3.3.4, 1.13.11.1, 1.13.11.3, 1.14.13.2, 1.14.12.10, 5.5.1.1, 1.14.13.1, 5.5.1.2, 5.3.2.6, 3.1.1.24, 1.2.1.16, 5.2.1.2, 2.6.1.57, 1.13.11.5, 2.3.1.16, 1.13.11.27, 2.6.1.9, 2.3.1.-,	NA

			2.3.1.9, 4.2.1.17	
		Unclassified Pseudomonadales	**3.1.2.23, 3.1.1.24, 5.2.1.2, 2.6.1.57, 4.1.1.44, 2.6.1.1, 1.13.11.5, 1.2.1.16,** 1.13.11.27, 2.3.1.9, 2.6.1.9, 1.1.1.157, 4.2.1.17, 2.3.1.-, 2.3.1.16	Tyrosine and Benzoate degradation
		Unclassified Pseudomonadaceae	**3.7.1.9, 1.14.12.-, 3.1.2.23, 3.1.1.24, 1.14.14.9, 1.13.11.15, 5.3.3.10,** 2.6.1.57, 1.14.14.9, 4.1.1.68, 5.2.1.2, 1.14.12.10, 4.1.1.44, 1.3.1.25, 1.14.12.10, 1.13.11.27, 1.13.11.5, 5.3.3.4, 2.6.1.1, 1.13.11.1, 2.3.1.9, 1.2.1.16, 1.2.1.60, 2.6.1.9, 1.13.11.3, 1.1.1.157, 2.3.1.174, 1.14.13.2, 1.14.12.10, 4.2.1.17, 5.5.1.1, 2.3.1.16, 5.5.1.2, 1.4.3.4, 2.3.1.-, 3.7.1.2, 5.3.2.6, 1.14.13.1, 4.1.3.39, 1.2.1.85, 4.1.3.17, 1.13.11.2, 4.2.1.80, 1.2.1.10, 1.13.11.8, 4.1.1.77	PAH, Tyrosine and Benzoate degradation
		Pseudomonas	**1.14.14.9, 1.13.11.15, 1.14.14.9, 4.1.1.68, 5.3.3.10, 1.14.12.10, 1.3.1.25, 1.14.12.10, 3.1.2.23, 1.13.11.3, 2.3.1.174, 1.14.13.2, 1.2.1.60, 5.3.3.4, 3.1.1.24, 1.13.11.1, 2.6.1.57, 1.14.12.10, 5.5.1.2, 1.13.11.27, 5.2.1.2, 3.7.1.2, 4.1.1.44, 5.5.1.1, 1.13.11.5, 1.1.1.157, 2.3.1.9, 5.3.2.6, 2.6.1.9, 2.6.1.1, 1.4.3.4, 2.3.1.16, 4.2.1.17, 1.2.1.16,** 4.1.3.39, 4.2.1.80, 2.3.1.-, 1.2.1.10, 1.14.12.-, 1.13.11.2, 1.14.13.1, 4.1.1.77	Tyrosine and Benzoate degradation
**3**	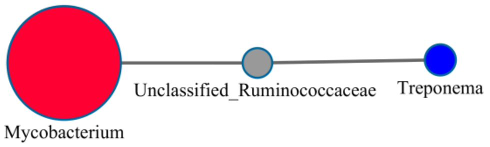	Mycobacterium	**1.13.11.-, 4.1.2.-, 1.13.11.38, 1.4.3.21, 1.2.1.10, 1.14.13.1, 4.1.3.39, 4.1.1.77, 1.4.3.4, 4.2.1.17, 4.1.1.46, 1.2.1.-, 2.3.1.16, 4.1.1.44, 2.3.1.9, 2.3.1.-, 4.2.1.80, 5.5.1.2, 4.1.2.34, 1.13.11.37, 1.1.1.157, 1.13.11.5, 1.2.1.16,** 2.6.1.9, 1.1.1.90, 2.8.3.6, 1.13.11.2, 1.13.11.4, 5.3.3.4, 1.13.11.1,	PAH, Naphthalene, Tyrosine and Benzoate degradation

			1.14.14.9, 1.14.12.10, 3.1.1.24, 3.1.2.23, 5.3.2.6, 1.13.11.3, 1.14.12.10, 1.13.11.27, 2.6.1.1, 5.3.2.6, 4.1.1.77, 2.3.1.-, 2.6.1.9, 2.3.1.9, 4.2.1.17, 4.1.1.44, 1.2.1.16, 2.3.1.16	
**4**	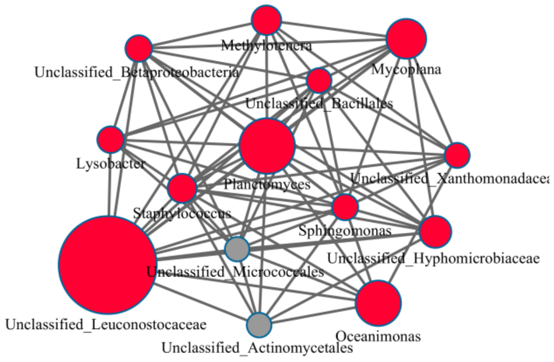	Planctomyces	**1.2.1.5,** 5.5.1.1, 5.3.2.6, 2.6.1.1, 2.3.1.-, 2.6.1.9, 2.3.1.9, 4.2.1.17	NA
		Oceanimonas	**3.7.1.5, 2.6.1.1, 1.13.11.4, 1.14.12.10, 1.3.1.25, 1.14.12.10, 5.3.3.4, 1.13.11.1, 1.13.11.3, 1.14.13.2, 1.14.12.10, 5.5.1.1,** 1.14.13.1, 5.5.1.2, 5.3.2.6, 3.1.1.24, 1.2.1.16, 5.2.1.2, 2.6.1.57, 1.13.11.5, 2.3.1.16, 1.13.11.27, 2.6.1.9, 2.3.1.-, 2.3.1.9, 4.2.1.17	Tyrosine and Benzoate degradation
		Sphingomonas	**1.13.11.14,** 1.13.11.37, 1.14.12.-, 6.2.1.25, 5.5.1.1, 1.2.1.85, 4.1.3.17, 1.13.11.4, 5.3.2.6, 1.14.12.10, 5.2.1.2, 1.13.11.1, 1.3.1.25, 2.6.1.1, 1.13.11.5, 1.13.11.27, 1.13.11.2, 1.1.1.157, 1.2.1.16, 2.8.3.6, 4.1.1.44, 4.2.1.80, 1.2.1.10, 3.7.1.2, 4.2.1.17, 2.3.1.9, 1.13.11.2, 2.6.1.9, 2.3.1.16, 4.1.1.68, 3.1.1.24, 4.1.1.77, 4.1.3.39, 4.2.1.83, 5.3.3.4, 2.6.1.57, 1.2.1.60, 1.13.11.3, 2.3.1.174, 1.14.12.10, 5.3.3.10, 5.5.1.2, 3.1.2.23	Benzoate degradation
		Staphylococcus	1.13.11.2, 5.3.2.6, 1.2.1.16, 2.6.1.9, 1.1.1.157, 2.3.1.-, 2.3.1.16, 2.3.1.9, 1.13.11.15, 1.14.14.9, 1.2.1.60, 1.14.13.2, 2.6.1.1, 3.1.1.24, 1.4.3.4, 4.1.1.77, 4.1.1.44, 4.2.1.17	NA
		Mycoplana	**1.1.1.312, 3.1.1.57, 4.1.3.17,** 4.2.1.83, 1.13.11.8, 1.14.13.2, 5.3.3.10, 1.4.3.4, 4.2.1.80, 5.2.1.2, 2.6.1.1, 1.13.11.5, 1.2.1.16, 1.13.11.27, 2.3.1.9, 1.1.1.157, 4.2.1.17, 2.6.1.9, 4.1.1.44, 2.3.1.16	Benzoate degradation
		Unclassified	**1.14.12.12, 1.13.11.2, 2.3.1.174, 1.13.11.3, 1.14.13.2,**	Naphthalene,

	Xanthomonadaceae	**4.1.1.68,** 5.2.1.2, 4.2.1.80, 2.6.1.57, 5.5.1.2, 1.13.11.5, 1.13.11.27, 3.1.1.24, 2.6.1.9, 4.1.1.44, 1.2.1.60, 2.3.1.-, 1.2.1.16, 2.3.1.16, 2.3.1.9, 2.6.1.1, 4.2.1.17	Tyrosine and Benzoate degradation
	Unclassified Hyphomicrobiaceae	**1.3.7.8, 1.1.1.368, 3.7.1.-, 4.2.1.100,** 6.2.1.25, 4.1.1.28, 4.1.2.45, 1.13.11.2, 3.1.1.57, 4.1.3.17, 1.14.13.1, 1.14.12.-, 5.3.2.6, 1.13.11.1, 1.13.11.2, 1.13.11.15, 2.6.1.1, 1.2.1.16, 2.8.3.6, 3.1.1.24, 2.3.1.-, 1.1.1.157, 1.2.1.60, 4.1.1.44, 2.3.1.9, 1.13.11.3, 1.14.14.9, 5.99.1.4, 5.2.1.2, 1.4.3.4, 1.14.13.2, 2.6.1.9, 4.2.1.17, 3.1.2.23, 1.13.11.27, 5.3.3.10, 5.5.1.2, 5.5.1.1, 2.3.1.16, 1.13.11.5	Benzoate degradation
	Unclassified Bacillales	1.13.11.2, 1.14.14.9, 5.3.2.6, 5.3.3.10, 2.3.1.-, 1.2.1.60, 4.2.1.80, 1.2.1.10, 4.1.1.77, 4.1.3.39, 2.6.1.9, 1.1.1.157, 1.13.11.5, 2.3.1.16, 1.13.11.27, 2.3.1.9, 1.2.1.16, 4.2.1.17, 1.4.3.4, 3.1.1.24, 2.6.1.1, 4.1.1.44	NA
	Unclassified Leuconostocaceae	5.3.2.6, 4.1.1.44, 2.3.1.-, 2.3.1.9, 1.1.1.157	NA
**5**	Kaistobacter	**1.13.11.14, 1.14.12.-, 1.13.11.37, 1.13.11.4,** 1.14.12.10, 1.3.1.25, 1.13.11.1, 6.2.1.25, 1.2.1.85, 1.13.11.2, 5.5.1.1, 4.1.3.17, 4.2.1.80, 1.2.1.10, 3.7.1.2, 4.1.1.68, 3.1.1.24, 5.3.2.6, 4.1.1.77, 4.1.3.39, 1.2.1.16, 5.2.1.2, 1.1.1.157, 2.6.1.57, 2.6.1.1, 4.2.1.17, 1.13.11.5, 2.8.3.6, 1.13.11.27, 2.6.1.9, 2.3.1.9, 1.13.11.2, 4.1.1.44, 2.3.1.16, 4.2.1.83, 5.3.3.4, 1.2.1.60, 1.13.11.3, 2.3.1.174, 1.14.12.10, 5.3.3.10, 5.5.1.2, 3.1.2.23	PAH, Tyrosine and Benzoate degradation
	Alicyclobacillus	**1.13.11.2, 1.14.14.9,** 1.13.11.4, 1.2.1.60, 4.1.1.68, 5.3.2.6, 1.2.1.10, 4.1.1.77, 1.1.1.157, 4.1.3.39, 2.6.1.1, 1.13.11.5,	Tyrosine and Benzoate

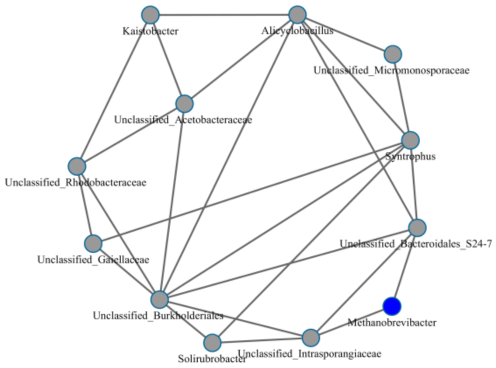		4.2.1.17, 2.3.1.-, 1.2.1.16, 2.3.1.16, 2.3.1.9, 2.6.1.9	degradation
	Unclassified Micromonosporaceae	**1.2.1.85,** 4.1.3.17, 4.1.1.77, 4.1.1.28, 1.2.1.10, 4.1.3.39, 3.7.1.2, 5.5.1.2, 1.1.1.157, 1.4.3.4, 1.13.11.27, 1.4.3.21, 1.2.1.16, 1.13.11.15, 2.6.1.9, 1.13.11.5, 2.3.1.16, 2.3.1.9, 4.2.1.17, 1.1.1.90, 1.13.11.3, 1.14.13.2, 1.14.12.10, 4.1.1.44, 2.3.1.-, 3.1.1.24	Benzoate degradation
	Unclassified Rhodobacteraceae	**6.2.1.27, 1.14.13.12, 4.1.1.28,** 4.1.3.17, 1.13.11.2, 1.1.1.312, 3.1.1.57, 1.14.13.1, 1.13.11.3, 1.14.12.-, 1.14.13.2, 1.13.11.15, 1.2.1.60, 5.5.1.2, 3.1.1.24, 3.7.1.2, 5.3.3.10, 2.6.1.1, 4.1.1.44, 2.6.1.57, 1.4.3.4, 1.2.1.16, 5.2.1.2, 1.13.11.27, 2.3.1.-, 1.13.11.4, 2.6.1.9, 1.13.11.8, 1.14.12.12, 4.2.1.17, 1.13.11.5, 2.3.1.9, 1.1.1.157, 1.13.11.2, 5.3.2.6, 4.1.3.39, 3.1.2.23, 4.2.1.80, 4.1.1.46, 1.13.11.1, 5.3.3.4, 2.3.1.16, 5.5.1.1	Tyrosine and Benzoate degradation
	Unclassified Acetobacteraceae	**1.13.11.37,** 2.8.3.6, 1.13.11.3, 1.14.13.2, 2.6.1.1, 5.5.1.2, 3.7.1.2, 5.3.2.6, 3.1.1.24, 1.4.3.4, 4.1.1.44, 2.3.1.-, 5.2.1.2, 1.2.1.16, 1.13.11.27, 4.2.1.17, 1.1.1.157, 2.3.1.16, 2.6.1.9, 2.3.1.9	Benzoate degradation

Supplemental Table S6. 16S rRNA gene sequence identities of isolates from different treatments.

**Bacterial isolates**	**Sources**	**Closed cultured organism (GenBank accession no.)**	**Phylum**	**Identity**
Tet-2	tetradecan	*Rhodococcus equi* strain SW9 (KF873018)	Actinobacteria	99%
Tet-3	tetradecan	*Brucella* sp. BS33 (HM132105)	Alphaproteobacteria	99%
Hex-2	hexacosane	*Nocardia* sp. NN256 (GU723668)	Actinobacteria	98%
hex-3	hexacosane	*Rhodococcus* sp. B5 (JQ773352)	Alphaproteobacteria	99%
hex-4	hexacosane	*Rhodococcus ruber* (JF895525)	Alphaproteobacteria	98%
pri-4	pristane	*Nocardia* sp. WT7 (KJ400422)	Actinobacteria	98%
pri-5	pristane	*Nocardia asteroides* strain APN00071 (KC262095)	Actinobacteria	98%
phe-2	phenanthrene	*Rhizobium* sp. HXG-C4 (GU257956)	Alphaproteobacteria	100%
phe-3	phenanthrene	*Marinomonas* sp. D6084 (DQ480145)	Gammaproteobacteria	99%

phe-4	phenanthrene	*Sinorhizobium* sp. R2A 23-7 (EF486317)	Alphaproteobacteria	98%
phe-5	phenanthrene	*Mycobacterium gilvum* Spyr1 (NR074644)	Actinobacteria	98%
pyr-1	pyrene	*Pseudomonas* sp. BC045 (HQ105013)	Gammaproteobacteria	99%
pyr-2	pyrene	*Gordonia* sp. OB51 (JN942152)	Actinobacteria	98%
pyr-3	pyrene	*Aminobacter anthyllidis* strain STM4645 (NR108530)	Alphaproteobacteria	98%
pyr-4	pyrene	*Stappia* sp. F2 (JF899875)	Alphaproteobacteria	98%
Pyr-5	pyrene	Sinorhizobium sp. R2A 23-7 (EF486317)	Alphaproteobacteria	99%
pyr-6	pyrene	*Mycobacterium* sp. JL852 (DQ985069)	Actinobacteria	99%
pyr-7	pyrene	*Mesorhizobium* sp. T61 (EU874890)	Alphaproteobacteria	99%
